# Amniotic microvesicles impact hatching and pregnancy percentages of *in vitro* bovine embryos and blastocyst microRNA expression versus *in vi*vo controls

**DOI:** 10.1038/s41598-019-57060-z

**Published:** 2020-01-16

**Authors:** Anna Lange-Consiglio, Barbara Lazzari, Flavia Pizzi, Antonella Idda, Fausto Cremonesi, Emanuele Capra

**Affiliations:** 1 Università degli Studi di Milano, Dipartimento di Medicina Veterinaria, Milano, Italy; 2 Centro Clinico-Veterinario e Zootecnico-Sperimentale di Ateneo, Università degli Studi di Milano, Milano, Italy; 3 Istituto di Biologia e Biotecnologia Agraria, Consiglio Nazionale delle Ricerche IBBA CNR, Lodi, Italy

**Keywords:** Biological techniques, Animal biotechnology

## Abstract

Embryo development and implantation are dynamic processes, responsive to external signals, and can potentially be influenced by many environmental factors. The aims of this study were to evaluate the effects of a culture *medium* supplemented with amniotic-derived microvesicles (MVs) on *in vitro* embryo hatching after cryopreservation, and pregnancy rate following embryo transfer. In addition, miRNA profiling of blastocysts produced *in vitro*, with or without (control; CTR) amniotic MV supplementation, was also evaluated using blastocysts produced *in vivo*. *In vitro* embryos were cultured with and without amniotic MV supplementation. *In vivo* blastocysts were obtained from superovulated cows. Samples for RNA isolation were obtained from three pools of 10 embryos each (*in vivo*, *in vitro*-CTR and *in vitro* + MVs). Our results show that the hatching percentage of cryopreserved *in vitro* + MVs embryos is higher (P < 0.05) than *in vitro*-CTR embryos and the pregnancy rate with fresh and cryopreserved *in vitro* + MVs embryos is higher than *in vitro*-CTR embryos. In addition, the analysis of differently expressed (DE) microRNAs showed that embryos produced *in vivo* are clearly different from those produced *in vitro*. Moreover, *in vitro*-CTR and *in vitro* + MVs embryos differ significantly for expression of two miRNAs that were found in higher concentrations in *in vitro*-CTR embryos. Interestingly, these two miRNAs were also reported in degenerated bovine embryos compared to good quality blastocysts. In conclusion, MV addition during *in vitro* production of embryos seems to counteract the adverse effect of *in vitro* culture and partially modulate the expression of specific miRNAs involved in successful embryo implantation.

## Introduction

In the mammalian reproductive tract, the oviduct secretes a variety of growth factors and cytokines, which may play an essential role in the development of initial stages of pre-implantation embryos^1^. The absence of these maternal-embryo signals could be an important cause of the poor quality of *in vitro* produced embryos (IVP), compared to those collected *in vivo*^2,3^. To mimic the *in vivo* crosstalk between oviduct and embryo and to improve the quality of *in vitro* produced embryos, co-culture systems with somatic cells have been widely used to increase blastocyst percentage and quality of the resulting embryos, and the induction of specific transcriptomic changes^4^.

Unfortunately, concern regarding viral transmission has resulted in the elimination of co-culture and the development of improved culture methods characterized by definite *medium* as synthetic oviductal fluid with amino acids (SOFaa^5^). However, the maternal-embryo communication *in vivo* and the communication between monolayer (‘helper’) cells and embryos *in vitro*, take place not only through soluble factors (growth factors, receptors and binding proteins) secreted by cells and embryos in the *medium*^6,7^ but also from insoluble factors. Recent studies have demonstrated that some molecules, including mRNA fragments and microRNAs (miRNAs), cannot freely cross the membrane and are thus released into the extracellular vesicle (EV) before its release from the cells^8^. In addition to genetic material, EVs contain molecules such as cytoskeletal proteins, immunoregulator molecules, signal transduction molecules, tetraspanins, heat shock protein, lipid rafts, etc^9^. The EVs can be categorized as exosomes or microvesicles (MVs), depending on the mechanisms responsible for their biogenesis. Exosomes, derived from multivesicular bodies, are released after fusion with the plasma membrane and vary in size from 30 to 100 nm, while MVs are formed and shed directly from the plasma membrane and have a size between 100–1000 nm^10^. Extracellular vesicles can be detected in all body fluids as well as in culture *medium* collected from different cell lines^8^ and appear to be the major mechanism by which cells communicate with their environment.

Until now no one has studied the role of EVs in paracrine mechanisms *in vitro* however, it is important to develop an effective embryo culture *medium* that might eventually also improve the efficacy of human embryo culture programs.

To improve the quality of *in vitro* produced bovine embryos, in our previous study^11^ bovine embryos were co-cultured with endometrial or amniotic-derived EVs. Using Nanosight and transmission electron microscope evaluation, these EVs were identified as MVs, based on their size and biogenesis. During the co-culture, amniotic or endometrial derived-MVs labelled with PKH26 were internalized into bovine blastomeres but the amniotic derived MVs have a number of effects on the embryo: increase the number of cells constituting the inner cell mass, improving quality, increasing viability, increasing expression of *GPX1* gene (protective against lipid peroxidation) and reducing expression of *BAX* gene (involved in apoptosis) compared to endometrial MVs and control (CTR). The amniotic secretome appears to better support *in vitro* embryo growth than the endometrial secretome. This result was surprising but it is likely that endometrial cells de-differentiate during culture in monolayers by losing cell polarity, cell height, ciliation, secretory activity, and responsiveness to hormones^12–15^. In this way, the secretions produced by endometrial cells *in vitro* are different from the *in vivo* secretions. Moreover, it is probable that amniotic and endometrial secretomes contain different components attributable to the different age of this tissue (adult for endometrium and fetal for amnion) however, there is no data in the literature to support this hypothesis.

To further study the effects of amniotic-derived MVs on bovine embryos and to evaluate whether MVs may potentially influence embryo implantation, we analysed *in vitro* embryo hatching after cryopreservation and pregnancy rate following embryo transfer.

In addition, the profiles of microRNAs (miRNAs) were characterized in *in vitro* produced embryos with and without MV supplementation as well as in *in vivo* produced embryos. The miRNAs are non-coding RNA molecules of about 22 nucleotides in length that can modulate transcription/expression levels of many target genes^16,17^.

The regulatory properties of miRNAs and their roles in mammalian gametogenesis and signaling in the context of embryonic development and implantation is well documented^18,19^. In addition, miRNAs have been reported to serve as non-invasive biomarkers to assess preimplantation developmental competence and embryo selection^20^.

## Results

### Amniotic cell isolation and characterization by reverse transcription-PCR analysis

**Figure 1:**
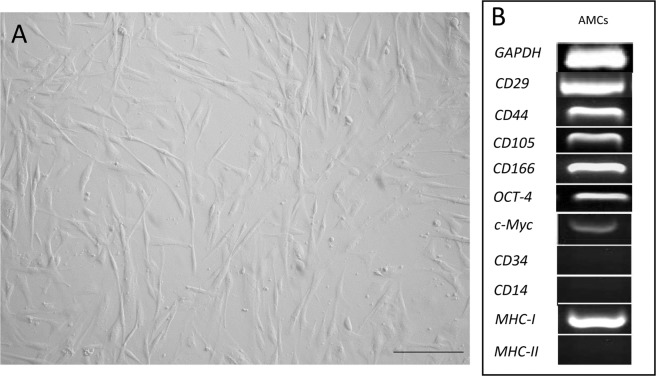
(**A**) Amniotic cell morphology. Magnification 20X, scale bar 20 µm. (**B**) RT-PCR analysis of mesenchymal (*CD29*, *CD44*, *CD105*, *CD166*), pluripotent (*Oct-4* and *c-Myc*) and haematopoietic (*CD34*, *CD14*) markers on AMCs at P3. Major Histocompatibility Complex (*MHC*) I and II gene expression is also reported.

Amniotic derived cells (AMCs) showed a typical stem cell phenotype (Fig. 1A) and expressed MSC (*CD29*, *CD44*, *CD105*, *CD166*) but not hematopoietic (*CD34* and *CD14*) markers. No expression of the Major Histocompatibility Complex, class II (*MHC-II*) was detected although *MHC-I* was found. Moreover, MSCs were found to express *Oct-4* and *c-Myc* (Fig. 1B*)*. These data are in concordance with those reported by Lange-Consiglio *et al*.^21^.

### EV identification

**Figure 2:**
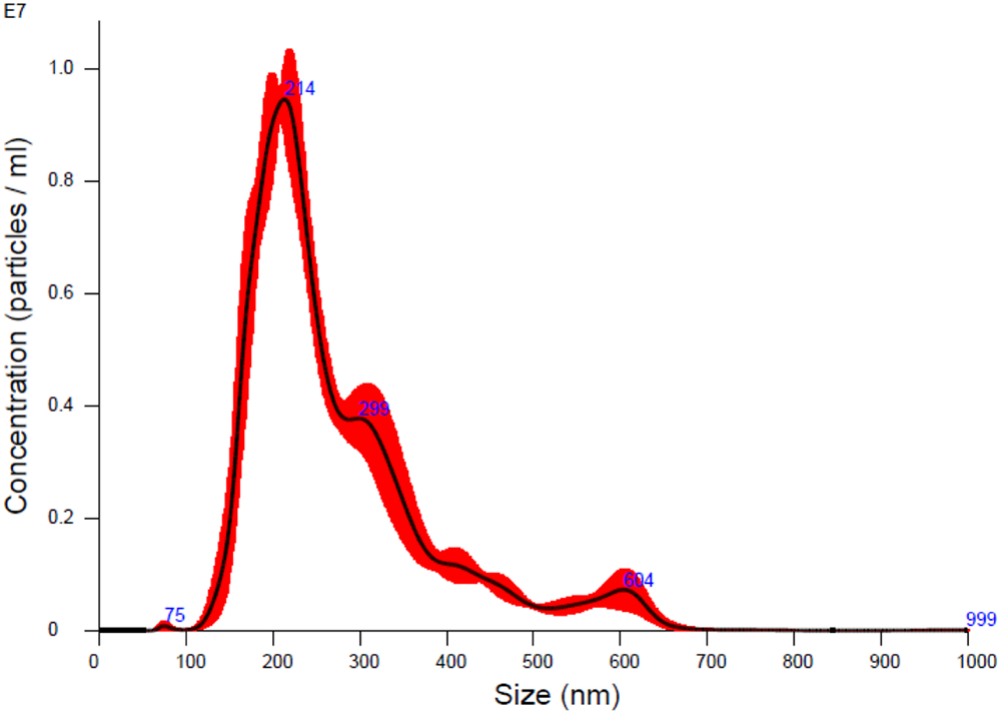
NanoSight analysis. Results from analysis of MVs purified from amniotic cells. Nanoparticle Tracking Analysis software allows the analysis of video images of the particle movement calculating the mean size and particle concentration values. The curve describes the relationship between particle number distribution (left Y-axis) and particle size (X-axis).

NanoSight analysis determined that amniotic EVs had a dimension between 75 nm and 700 nm, with a mean of 275 ± 8.4 nm (Fig. 2). These characteristics, classify these EVs as microvesicles (MVs).

### Percentage of *in vitro* produced embryos

A total of 3782 oocytes were fertilized over the course of 18 replicates. Embryo morphology was evaluated on day 7 after fertilization under a stereomicroscope (Leica microsystems, Milan, Italy) and the embryos were grouped according to their development stage (morula, compact morula, and blastocyst). Poor quality morulas/compact morulas were classified as degenerate if there was a loss of plasma membrane integrity (lysis) and/or generalized loss of cell forms.

The percentage of B7 was 34.59 ± 1.32% (709/2050) in *in vi*tro-CTR and 34.24 ± 1.71% (593/1732) in *in vitro* + MVs (P > 0.05) (Table 1).

**Table 1:** Data obtained under different experimental conditions *in vitro* and *in vivo*.

Experimental conditions	*In vitro*-CTR	*In vitro* + MVs
Rate of embryo production	(709/2050) 34.59 ± 1.32%^a^	(593/1732) 34.24 ± 1.71%^a^
Survival percentage after cryopreservation	(87/266) 32.71 ± 6.26%^a^	(78/181) 43.09 ± 5.73%^b^
Pregnancy rate after fresh embryo transfer (D28)	(11/30) 36.67%^a^	(20/30) 66.67%^b^
Pregnancy rate after cryopreserved embryo transfer (D28)	(3/30) 10%^a^	(11/30) 36.67%^b^
Pregnancy rate after fresh embryo transfer (D70)	(10/30) 33.33%^a^	(20/30) 66.67%^b^
Pregnancy rate after cryopreserved embryo transfer (D70)	(2/30) 6.67%^a^	(11/30) 36.67%^b^

Different small letters superscript (a,b) in the same line indicate statistically different comparisons (p < 0.05) between *In vitro*-CTR and *In vitro* + MVs.

### Survival percentage after cryopreservation

After cryopreservation, embryo survival, in terms of hatching blastocysts, was statistically different (P < 0.05) between *in vitro*-CTR (32.71 ± 6.26%; 87/266) and *in vitro* + MVs (43.09 ± 5.73%; 78/181), respectively (Table 1).

### Percentage of pregnancy after embryo transfer

On day 28 (D28), the pregnancy rate was statistically different (P < 0.05) after embryo transfer of fresh embryos, 36.67% (11/30 cows) for *in vitro*-CTR and 66.67% (20/30 cows) for *in vitro* + MVs. There was also a statistically significant difference (P < 0.05) in pregnancy rate for transfer of cryopreserved embryos: 10% (3/30) for *in vitro*-CTR embryos versus 36.67% (11/30) for *in vitro* + MV (Table 1). On day 70 (D70), the pregnancy rate decreased to 3.34% for both fresh and cryopreserved *in vitro*-CTR embryos, while for *in vitro* + MVs embryos no late embryo mortality was recorded for either fresh or cryopreserved embryos (Table 1).

### *In vivo* embryo production

After superovulation in three cows, 34 embryos were collected, with an average of 11.36 ± 1.42 embryos.

### miRNA data analysis

Nine samples were sequenced, each from a pool of 10 embryos, belonging to three groups (*in vivo*, i*n vitro*-CTR and *in vitro* + MVs) (see Supplementary File S1 for statistics).

About 35 million reads were sequenced for each condition and replicate, 1% of which were assigned to miRNAs. In total, 294 miRNAs were identified at least in triplicate in one group (197 Bos taurus bta-miRNAs, 52 novel and 45 novel homologous to related species).

**Figure 3:**
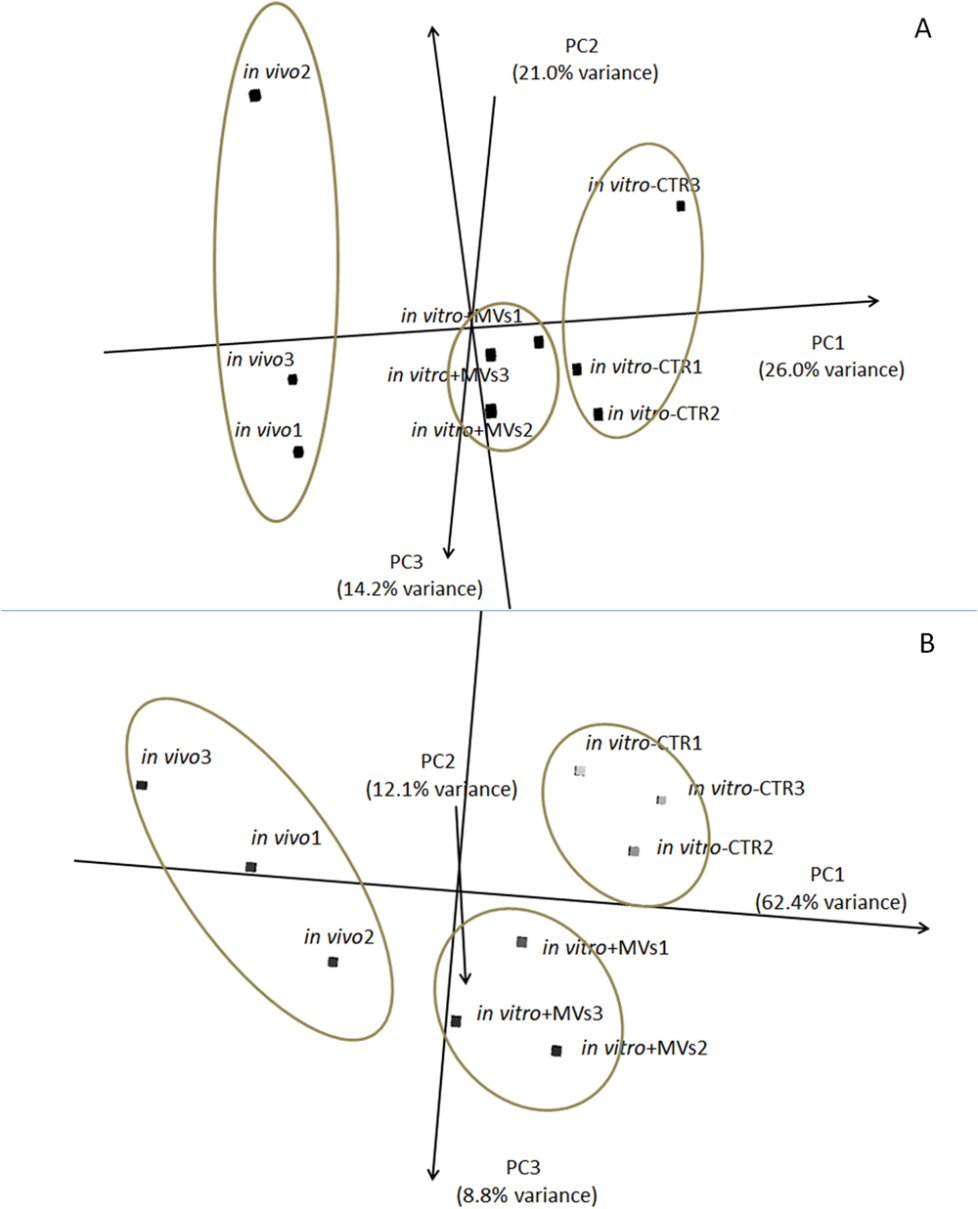
Principal component analysis showing: (**A**) the 294 miRNAs present at least in triplicate in one condition, (**B**) the 34 DE-miRNAs.

Principal Component Analysis (PCA) on these miRNAs showed that *in vivo* produced embryos were clearly different from *in vitro* produced embryos (PC1). The two groups of embryos obtained following *in vitro* culture clustered close to one another, but the *in vitro* + MVs group was closer to *in vivo* samples (Fig. 3A).

**Figure 4:**
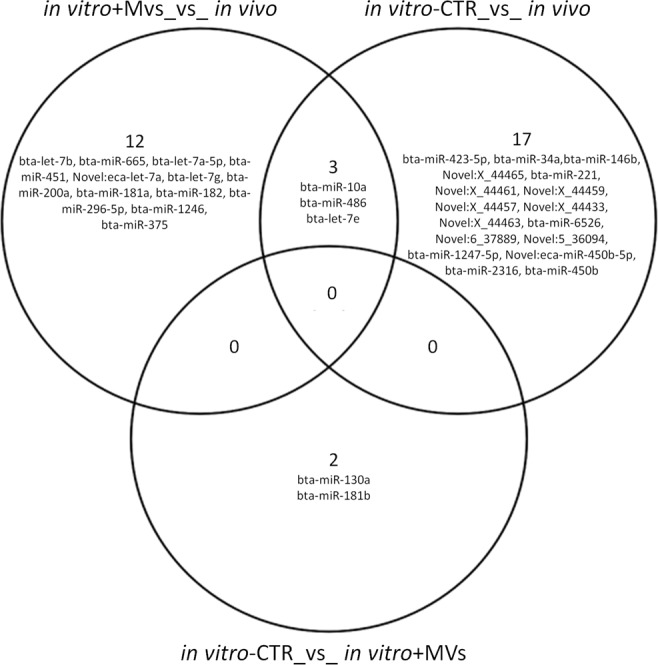
Venn Diagram of the differentially expressed (DE)-miRNAs for the three comparisons (*in vivo* vs *in vitro* + MVs, *in vivo* vs *in vitro*-CTR, *in vitro* + MVs vs *in vitro*-CTR).

The number of miRNAs found to be differentially expressed ((DE)-miRNAs) in three comparisons (*in vivo* vs *in vitro* + MVs, *in vivo* vs *in vitro*-CTR, *in vitro*-CTR vs *in vitro* + MVs) were 15, 20 and 2, respectively (False Discovery Rate (FDR) < 0.05) (Supplementary File S2). PCA calculated on DE-miRNAs showed a separation of the three groups with a distinctive miRNA trait (Fig. 3B). PC1, which explains 62.4% of the variance, clearly separates *in vivo* and *in vitro* produced embryos even if MV addition seems to ameliorate the effect of *in vitro* production. Three DE-miRNAs (bta-miR-10a, bta-miR-486 and bta-let7-e) are shared among the *in vivo* and the two *in vitro* groups (Fig. 3). Finally, the expression of two miRNAs (miR 130a, miR-181b) differed significantly between *in vitro* embryos obtained with or without MVs (Fig. 4).

### miRNA validation by quantitative polymerase chain reaction (q-PCR)

Five miRNAs (bta-let-7a-5p, bta-miR-130a, bta-miR-34a, bta-miR-423-5p, bta-miR-486) were validated by q-PCR using two reference smallRNAs (SNORD95 and RNU6). The fold change among three groups (*in vivo*, i*n vitro*-CTR and *in vitro* + MVs) were compared with those of miRNA sequencing (Supplementary File S3). qPCR confirmed smallRNA-seq pattern: bta-let-7a-5p was validated with both smallRNA references, whereas bta-miR-130a and bta-miR-34a were validated with RNU6 and bta-miR-423-5p, bta-miR-486 with SNORD95.

## Discussion

In recent years, the roles of EVs in biological processes such as implantation and embryo development^8^ have been widely investigated. The supplementation of *in vitro* embryo culture *medium* with bovine amniotic derived MVs has been shown to improve the quality of bovine embryos^11^. To understand the effects of amniotic-derived MVs on bovine embryos and their potential influence on embryo implantation, we performed a further *in vitro* and *in vivo* study. The results show that the hatching percentage of cryopreserved *in vitro* + MVs embryos is higher compared to the *in vitro*-CTR embryos. In addition, *in vivo*, the D28 recipient pregnancy rates are higher with both fresh and cryopreserved *in vitro* + MVs embryos compared *in vitro*-CTR embryos. At D70, late embryonic loss was found only in both fresh and cryopreserved *in vitro*-CTR embryos, while no late embryo mortality was recorded in *in vitro −* MV embryos. From these results, it can be concluded that the MVs had a positive effect on both the survival of frozen/thawed embryos and their implantation rates. We do not know how the MVs improve the implantation efficiency. It is likely that MV supplementation reduces apoptosis^11^ and degeneration and this assumption could explain the higher hatching rate in cryopreserved *in vitro* + MVs embryos and the higher recipient pregnancy rate *in vivo* with both fresh and cryopreserved *in vitro* + MVs embryos compared to *in vitro*-CTR embryos. Since it has recently been proposed that miRNAs could serve as molecular markers of blastocyst quality^20,22,23^, we wanted to investigate whether miRNAs could be related to the success of hatching and implantation. To this end, embryo miRNA profiling in the different embryo culture conditions (*in vitro*, with or without MVs, and *in vivo*) was performed.

It is known that messenger RNA and miRNA can be transferred from spermatozoa to the oocyte at fertilization^24^ and that altered spermatozoan mRNA profiles could influence *in vitro* fertilization^25^. In order to minimize the bull effect, ejaculate from a single bull of proven fertility was used.

To our knowledge, this is the first study reporting the complete miRNA profiling of blastocysts fertilized *in vitro* compared with those collected *in vivo*. Few studies have investigated the importance of specific miRNAs in successful embryonic development. For example, a consistent downregulation of miR-199-5p was seen in murine embryos fertilized *in vitro* compared with those fertilized *in vivo*^26^.

Our results show that miRNAs represent only a small proportion of the total small non-coding RNA (ncRNA) content in blastocysts. SmallRNA profiling of pools of 30 bovine blastocysts using deep sequencing showed that the vast majority of reads mapped to ncRNAs other than miRNAs, as previously demonstrated by Pasquariello *et al*.^27^. Similarly, only about 1% of the total reads sequenced in our experimental conditions was assigned to miRNAs.

In our work, the miRNA profiles of the three groups under study (*in vivo*, *in vitro*-CTR and *in vitro* + MVs) are different, and each group shows a distinctive miRNA trait. In the DE-miRNAs PCA, it is interesting to note that PC1, accounting for 62.4% of the variance, clearly separates *in vivo* and *in vitro* groups, and that three miRNAs were commonly up-regulated in *in vivo* vs *in vitro* produced embryos: let-7e, miR-10a and miR-486. miRNAs were also validated by qPCR.

It is reported that the let-7 family members are essential for correct blastocyst maturation^28^ and these are differently expressed in mouse uterus during the peri-implantation period^29^. Specifically, in rats, let-7a expression was observed to change temporally and spatially in the uterus, playing an important role in blastocyst implantation and decidualization and in the mutual relationship between the receptive uterus and competent blastocyst^29^. In addition, the miRNA let-7e is also involved in embryo implantation. However, there are conflicting data on the extent of expression of the let-7 family members, as downregulation in 5-aza-dC treated mouse pronuclear embryos (with inhibition of DNA methylation) alters embryo development^30^. On the other hand, the forced-expression of let-7a in dormant mouse blastocysts, activated by estradiol, reduces blastocyst attachment and outgrowth *in vivo* and *in vitro*, providing the first evidence that the let-7 family is involved in regulating the implantation process^31^. Our results show that let-7 family members are more strongly expressed in *in vivo*- than in *in vitro* produced blastocysts which contradicts the results of Liu *et al*.^31^ but it is likely that the expression of let-7a in murine blastocysts was excessively forced (about 100 times above physiological conditions), negatively affecting the implantation competency of the activated blastocysts.

MiR-10a is encoded in the Hox clusters upstream of *HoxB4* gene and has been shown to target several HOX transcripts, suggesting a co-regulation with the neighbouring Hox genes and a role as a developmental regulator^32^. In cultured embryos, ethanol treatment up-regulates miR-10a in mice and induces major fetal teratogenesis^33^. Mis-regulation of miR-10a caused by ethanol exposure is, therefore, lethal for the embryo and fetus. Interestingly, co-incubation with folic acid blocks ethanol-induced teratogenesis, down-regulates miR-10a and improves the blastocyst development^33^. Conversely, we found a higher level of miR-10a in *in vivo* vs *in vitro* blastocysts, suggesting that a minimum level of miR-10a is necessary for correct blastocyst maturation.

To date, there is no information in the literature regarding miR-486 apart from data concerning the significantly increased expression of miR-486-3p in the mouse placental villus during the peri-implantation period^34^. The higher expression of this miRNA in *in vivo* blastocysts compared to those produced *in vitro*, shown for the first time in our study, highlights the importance of this miRNA for correct blastocyst growth. These findings, however, need further investigation.

Our data show that *in vitro* embryo production modifies miRNA content in the blastocyst. The supplementation of culture *medium* with MVs seems to partially change the miRNA profile of *in vitro* + MVs embryos compared to *in vitro*-CTR embryos. Two miRNAs were significantly different between *in vitro* embryos obtained in the two conditions: miR-130a and miR-181b, which undergo up-regulation in *in vitro*-CTR embryos. These miRNAs were previously reported to regulate blastocyst development and embryo implantation. Mir-130a expression was observed to increase linearly during the 1–8 cell stage, but drastically decrease in the blastocyst, suggesting that miR-130a has a critical role in gene regulation in early bovine embryo development^18,35^.

MicroRNAs are also involved in fetal-maternal crosstalk. A uterine down-regulation of expression of miRNA-181 family members is associated with the onset of embryo implantation that occurs in mice on the evening of day 4-post coitus^36^. Our results suggest that these two miRNAs have to be down-regulated during embryo development. However, *in vitro* culture without MVs induces up-regulation of these two miRNAs. There was over expression of miR-181a in degenerate bovine embryos compared to healthy bovine embryos^37^ and mir-181b, and with miR-191-5p, were the miRNA most released into the conditioned *medium* by human embryos^38^. Our data on *in vitr*o-CTR embryos confirm this up-regulation and demonstrate that MV supplementation modifies miRNA expression ameliorating the quality of *in vitro* produced embryos.

## Conclusion

In conclusion, small RNA profiling showed a different miRNA expression between *in vivo* and *in vitro* produced blastocysts. This is likely to be due to epigenetic effects related to the different environmental culture conditions. Addition of amniotic MVs during *in vitro* embryo production seems to influence the developmental capacity and implantation potential of the embryos and regulate the expression of specific miRNAs (miR-130a and miR-181b) that regulate blastocyst development; these effects are involved in the success of embryo implantation, probably improving cross-talking between embryo and endometrium.

Further studies relating to other cargo components of the MVs are required, but in the meantime, these results could contribute to optimization of bovine embryo culture methods, and suggest new ways to improve human embryo culture technique.

## Materials and Methods

Chemicals were obtained from Sigma-Aldrich Chemical (Milan, Italy) unless stated otherwise, and tissue culture plastic dishes were purchased from Euroclone (Milan, Italy).

Ovaries were collected from Holstein Friesian cows slaughtered in a slaughterhouse (INALCA, Ospedaletto Lodigiano, Lodi, Italy) under national food hygiene regulations.

Allanto-amniotic membranes were obtained at term from normal pregnancies and parturitions from three Holstein Friesian cows (Bos taurus). All procedures were performed according to approved animal care and use protocols of the Università degli Studi di Milano ethics committee (OPBA 118_2017) and to good veterinary practice for animal welfare as to European directive 2010/63/UE. Moreover, written informed consent was obtained from farmers at the beginning of the study.

### Amniotic cell isolation and culture

Allanto-amniotic membranes were collected and processed following a standard protocol as previously reported^21^. Briefly, the allanto-amnion were kept at 4 °C and processed within 12 h. The amniotic membrane was separated from its juxtaposed allantois and cut into small pieces (about 9 cm^2^ each) that were digested with 0.93 mg/ml collagenase type I and 20 mg/ml DNAse (Roche, Mannheim, Germany) for approximately 3 h at 38.5 °C. Debris were removed using a 100 mm cell strainer and mobilized cells were collected by centrifugation at 200 × g for 10 min. Amniotic cells were cultured in a *medium* composed of HG-DMEM supplemented with 10% fetal calf serum (FCS), 1% penicillin (100 UI/mL)–streptomycin (100 mg/mL), 0.25 mg/mL amphotericin B and 2 mM L-glutamine at 38.5 °C in a humidified atmosphere of 5% CO_2_ until passage (P) 3.

### Amniotic cell characterization by reverse transcription-PCR analysis

Some mesenchymal (*CD29*, *CD44*, *CD105*, *CD166*), pluripotent (*Oct-4* and *c-Myc*) and haematopoietic (*CD34*, *CD14*) markers were evaluated by RT-PCR analysis at P3. Expression of *MHC-I* and *MHC-II* was also evaluated. *GAPDH* was employed as a reference gene. Equine-specific oligonucleotide primers and conventional PCR were the same ones used for the standard characterization of these cells as reported by Corradetti *et al*.^21^.

### Microvesicle isolation

Amniotic cells at P3 at confluence were cultured for one night in a serum-free *medium* (Ultraculture, Lonza, Milan, Italy). The microvesicle isolation was performed according to the protocol of Perrini *et al*.^11^. Briefly, the culture *media* from three amniotic membranes was collected, pooled, and centrifuged at 3500 × g for 20 min to remove cellular debris. Then, MVs were obtained by ultra-centrifuging the pooled *medium* at 100,000 × g for 1 h at 4 °C (Beckman Coulter Optima L - 100 K). The pellet was washed in serum-free *medium* 199 containing N-2-hydroxyethylpiperazine-N-2-ethanesulfonic acid (HEPES) 25 mM and ultra-centrifuged again under the same conditions. The pellet of MVs was split for MV analysis or use in the *in vitro* study.

### Measurements of MVs

As described by Bruno *et al*.^39^, size and concentration of MVs were evaluated by the NanoSight LM10 instrument (Nanoparticle tracking analysis, NTA, Nano-Sight Ltd., Amesbury, U.K.), which permits discrimination of microparticles less than 1 µm in diameter.

### *In vitro* embryo production

The *in vitro* embryo production is a standard protocol composed of three steps: collection of oocytes and their *in vitro* maturation, *in vitro* fertilization, and *in vitro* culture of embryos. In our laboratory, these steps are standardized and performed according to the protocol of Perrini *et al*.^11^ and Lange-Consiglio *et al*.^40^.

#### Collection of oocytes and *in vitro* maturation (IVM)

Ovaries were collected from slaughtered Holstein-Friesian cows (Bos Taurus) with unknown history (age, genealogy, and physiological status). Ovaries were transported to the laboratory in sterile saline solution (0.9% NaCl) supplemented with 150 mg/L kanamycin and maintained at 30 °C. Oocytes were retrieved by aspiration of 3–5 mm diameter follicles with 18 G needles. Cumulus–oocyte complexes (COCs) were selected and washed three times in pre-incubated TCM 199-Hepes buffered supplemented with 10% FCS.

*In vitro* maturation was performed under standard condition for 24 h in TCM 199 Earl’s Salt *medium* supplemented with 10% FCS, 5 μg/mL LH (Lutropin, Vetoquinol, France), 0.5 μg/mL FSH (Folltropin, Vetoquinol), 0.2 mM sodium pyruvate, 5 μg/mL gentamycin and 1 mg/mL estradiol 17β. Cultures were performed in 70 μL droplets (up to 20 oocytes/droplet) of the *medium* under mineral oil, at 38.5 °C in 5% CO_2_.

#### *In vitro* fertilization (IVF)

*In vitro* fertilization was performed in Tyrode’s-albumin-lactate-pyruvate (TALP) *medium* containing 2 mM penicillamine, 1 mM hypotaurine, 250 mM epinephrine, 20 μg/mL heparin, 114 mM NaCl, 3.2 mM KCl, 0.4 mM NaH2PO4, 10 mM sodium lactate, 25 mM NaHCO3, 0.5 mM MgCl2-6H20, 2.0 mM CaCl2-2H2O, 6 mg/mL bovine serum albumin (BSA,), 5 μl/mL gentamicin, 0.2 mM sodium pyruvate. Frozen-thawed semen from a single bull of proven fertility was prepared by Percoll density gradient (Amersham Pharmacia Biotec) (45/90%). Semen was thawed at 37 °C for 30 s, placed on the top of the Percoll gradient and centrifuged for 30 min at 300 × g. The semen suspension was diluted in the appropriate volume of fertilization *medium* to obtain a final concentration of 10^7^ spermatozoa per mL. An aliquot of 10 µL of semen was co-incubated with matured oocytes for 18 h at 38.5 °C in 5% CO_2._ Cultures were performed in 70 μl droplets (up to 20 oocytes/droplet) of the *medium* under mineral oil. At the end of gamete co-culture, cumulus cells were completely removed and cumulus-free presumptive zygotes were randomly transferred into different culture systems and cultured up to day 7.

#### *In vitro* culture (IVC)

The standard *medium* for IVC was synthetic oviductal fluid with amino acids (SOFaa; Holm *et al*. 1999) composed of 1.1 M NaCl, 72 mM KCl, 12 mM KH_2_PO_4_, 7.4 mM MgSO_4_, 50 mM DL-lactate, 250 mM NaHCO_3_, 260 mM phenol red, 100 mM sodium pyruvate, 178 mM CaCl_2_-2H_2_O, 125 mM Hepes sodium salt, 30.8 mM glutamine, 500 mM glycine, 84.2 mM alanine, 100X MEM non-essential, 100X BME, 2.8 mM Myo-Inositol, 340 mM trisodium citrate, 2% FCS, 0.005 gr/mL BSA, 0.2 mM sodium pyruvate, 5 µL/mL gentamicin.

At the beginning of the culture in SOFaa, presumptive zygotes were randomly assigned to a control group (CTR, no supplementation), or to group in which SOFaa was supplemented with 100 × 10^6^ EVs/ml as previously studied by Perrini *et al*.^11^ after dose-response curve.

*In vitro* culture was performed for 7 days in 5% O_2,_ 5% CO_2_ and 90% N_2_ in a humidified atmosphere at 38.5 °C. Cultures were performed in 70 μl droplets (up to 20 oocytes/droplet) of the *medium*. In the standard protocol, the *medium* is renewed on days 3 and 6 during the culture period. In this study, to avoid stress to the embryos and to allow the action of MVs, the *medium* was renewed on days 3 and 5, and the day fifth was chosen to add MVs to SOF^11^. SOF only was also added to the control group on the same days.

On day 7, blastocysts from the control and MV groups were retrieved from the IVC drops. One set of grade 1 blastocysts, according to International Embryo Technology Society^41^ classification system, either fresh or cryopreserved was used for embryo transfer. Another set (ten blastocysts for each group) was washed in sterile PBS and immediately cryopreserved in liquid nitrogen for genomic study.

### Cryopreservation of blastocysts and warming

Excellent quality blastocysts collected on day 7 (B7) were cryopreserved with ethylene glycol 1.5 M using a standard slow freezing curve. Thawing was accomplished by removing the cryoprotectant by washing in PBS supplemented with 0.2% BSA. Embryos were cultured in SOFaa and their *in vitro* survival and growth were evaluated for the 3 days before hatching.

### Recipient animals

One hundred and twenty Holstein-Friesian cows were enrolled in this study as recipients of an *in vitro* produced embryo. Written informed consent was obtained from the owners and the cows were used in accordance to the approved ethics application. No adverse outcomes were observed during synchronization, embryo transfer or pregnancy.

### Synchronization of oestrus, embryo transfer and pregnancy diagnosis

Recipient heifers were synchronized by a luteolytic dose of Prostaglandin PGF2-α (Estrumate, Schering Plough Animal Health, Segrate, MI, Italia) so that they displayed oestrus on the same day in which the *in vitro* insemination was performed (termed day 0). Only animals with a clinically detectable and well-developed corpus luteum (CL), received one fresh or one cryopreserved *in vitro*-CTR embryo or one fresh or cryopreserved *in vitro* + MV embryo 7 days after oestrus. Embryo transfer was performed into the uterine horn ipsilateral to the CL. On day 28 after transfer, and on day 70, pregnancy status was established by ultrasound.

### *In vivo* embryo production

Three Holstein-Friesian cows, 3 to 4 years old, with a history of normal fertility, were enrolled in this study. After detection of a well-developed CL, the animals were treated with a luteolytic PGF2-α dose and, nine days after induced estrus, the cows were superovulated with a combination of FSH and LH (Pluset, Calier SA, Barcelona, Spain) at the total dose of 1,000 U.I. in ten decreasing doses for 5 days (3, 3, 2.5, 2.5, 2, 2, 1.5, 1.5, 1, 1 ml). The cows were inseminated twice, at 12 hour interval, starting 12 h after onset of oestrus, with the same cryopreserved semen. Seven days after artificial insemination, embryos were collected under epidural anesthesia by procaine hydrochloride (IZO, Brescia, Italy). The uteri were non-surgically flushed with Ringer’s solution (Terumo, Tokyo, Japan) containing 0.1% fetal calf serum (FCS) through a multi-eye 16-French embryo collection catheter (Nipro, Osaka, Japan).

Collected embryos were counted and evaluated following the IETS criteria. Only grade 1 blastocysts were used for genomic study, after washing in sterile PBS and immediate cryopreservation in liquid nitrogen.

### RNA isolation

Samples for RNA isolation were obtained from pools of 10 embryos each for each condition (*in vivo*, *in vitro*-CTR and *in vitro* + MVs). Total RNA was isolated by NucleoSpin1 miRNA kit (Macherey-Nagel, Germany) following the protocol, in combination with TRIzol (Invitrogen, Carlsbad, CA, USA) lysis with large and small RNA in a single fraction (total RNA), with few modifications: the final step of RNA elution from column was performed using a minimum amount of nuclease free water (14 µl). Quality and concentration of RNA were determined using Agilent 2100 Bioanalyzer (Santa Clara, CA, USA). The isolated RNAs were stored at −80 °C until use.

### Library preparation and sequencing

Nine libraries were obtained working in triplicate for each condition (*in vivo*, *in vitro*-CTR and vitro + MVs). Libraries were prepared using the TruSeq Small RNA Library Preparation kit, according to manufacturer’s instructions (Illumina). Small non-coding RNA (snc-RNAs) libraries were pooled together and purified with Agencourt®AMPure® XP (Beckman, Coulter, Brea, CA) (1 Vol. sample: 1.8 Vol. beads) twice. Concentration and quality of libraries were assessed by Agilent 2100 Bioanalyzer prior sequencing on a single lane of Illumina Hiseq. 3000 (San Diego, CA, USA).

### miRNA data analysis

MiRNA analysis was performed as previously reported^42^: after quality checking with FastQC (http://www.bioinformatics.babraham.ac.uk/projects/fastqc/) and trimming with Trimmomatic^43^, Illumina sequences were input to miRDeep2^44^ for miRNA detection and discovery. Bos taurus miRNAs available at MirBase (http://www.mirbase.org/) were used to identify known miRNAs. Known miRNAs from related species (sheep, horse, and goat) available at MirBase were also input into miRDeep2 to support classification of novel miRNAs. The miRDeep2 quantifier module was used to quantify expression and retrieve counts for the detected known and novel miRNAs. Differential expression analyses between samples were run with the Bioconductor edgeR package (version 2.4) (false discovery rate [FDR] < 0.05)^45^. MicroRNA cluster analysis was performed with Genesis^46^.

### miRNA validation by quantitative polymerase chain reaction (q-PCR)

Samples of isolated RNA were pooled to obtain three samples representing each treatment (*in vivo*, i*n vitro*-CTR and *in vitro* + MVs) that were retro-transcribed using the miScript II RT Kit following the manufacturer’s instructions (Qiagen, Inc., Valencia, CA, USA). Quantitative real-time (qPCR) and reverse transcription polymerase chain reaction (RT-PCR) was carried out as previously reported^47^ with some modification. cDNAs were firstly preamplified using miScript preAMP kit (Qiagen, Inc.) following the manufacturer’s instructions except that miScript Primers were used instead of miScript PreAMP Primer Mix. Pre-amplification reactions were done in 10 µl volumes containing 5x miScript PreAMP Buffer (2 μl), HotStarTaq DNA Polymerase (0.8) μl, miScript Primer (0.4 μl), RNase-free water (4.4 μl), miScript PreAMP Universal Primer (0.4 μl), template cDNA (2 μl). Each amplified cDNA was quantified using QuantStudio 6 Flex Real-Time PCR System (Thermo Fisher Scientific, Waltham, MA, USA) with miScript SYBR Green PCR Kit (Qiagen, Inc.). Reactions were performed in 10 µl volumes containing 1 μl of each miscript Primer and 1 μl mL of universal reverse primer (Qiagen, Inc.), 2 μl cDNA, 1 μl RNase-free water and 5 μl QuantiTect SYBR Green PCR Master Mix according to the manufacturer’s protocols. The same specific miScript Primers were used in both pre-amplification and real-time reactions. The primers used for bta-let-7a-5p, bta-miR-130a, bta-miR-34a, bta-miR-423-5p, bta-miR-486 were Hs_let-7a_2, Hs_miR-130a_1, Hs_miR-34a_1, Hs_miR-423-5p_1, Hs_miR-486_1 miScript Primer Assay (Qiagen, Inc.), respectively. For the normalization, the references U6 small nuclear RNA Hs_RNU6-2_11 and SNORD95 small nucleolar RNA, C/D box 95 miScript Primer (Qiagen, Inc.) were used. Negative controls using water in place of sample were performed alongside each reaction. Reactions were run using the cycling parameters of 95 °C for 15 min, 40 cycles of 94 °C for 15 s, 55 °C for 30 s and 70 °C for 30 s. Relative expression levels for each treatment were calculated using the 2^−ΔΔCt^ method^48^.

### Statistical analyses

Statistical analyses were performed by ANOVA with non-parametric Kruskall-Wallis test and for pregnancy rate by chi square. Differences were considered statistically significant at P < 0.05.

## Supplementary information

Supplementary information.

## Data Availability

The datasets generated during and/or analysed during the current study are available from the corresponding author on request.
