# Supplementary material for: Amniotic microvesicles impact hatching and pregnancy percentages of in vitro bovine embryos and blastocyst microRNA expression versus in vivo controls
Source: Sci Rep. 2020 Jan 16;10:501. doi: 10.1038/s41598-019-57060-z (PMC6965648; doi:10.1038/s41598-019-57060-z)

Amniotic microvesicles impact hatching and pregnancy percentages of *in vitro* bovine embryos and blastocyst microRNA expression versus *in vi*vo controls

Anna Lange Consiglio[1,2], Barbara Lazzari[3], Flavia Pizzi[3], Antonella Idda[1], Fausto Cremonesi[1,2], Emanuele Capra[3]

Supplementary file S1. Statistics of miRNAs sequencing experiment for each condition (*in vitro*+MVs, *in vitro*-CTR and *in vivo*) in replicates (1, 2, 3).

	in vitro+ MVs1	*in vitro*+ MVs2	*in vitro*+ MVs3	*in vitro*-CTR1	*in vitro*-CTR2	*in vitro*-CTR3	*in vivo*1	*in vivo*2	*in vivo*3
reads after trimming	2,9E+07	1,7E+07	5,7E+07	3,1E+07	3,4E+07	5,1E+07	2,9E+07	7,9E+06	5,0E+07
miRNAs identified	2,8E+05	7,1E+04	3,3E+05	4,2E+05	1,6E+05	5,2E+05	2,1E+05	3,4E+04	2,8E+05
Percentage (%)	0,96	0,41	0,58	1,37	0,48	1,02	0,72	0,43	0,57

Supplementary file S2. Number of miRNAs found to be differentially expressed (DE-miRNAs) among three comparisons (*in vivo* vs *in vitro*+MVs, *in vivo* vs *in vitro*-CTR, *in vitr*o-CTR vs *in vitro*+MVs).

in vitro+MVs vs *in vivo*				*in vitro*-CTR vs *in vivo*				*in vitro*+MVs vs *in vitro*-CTR			
	logFC	PValue	FDR		logFC	PValue	FDR		logFC	PValue	FDR
bta-miR-10a	-3,9	2,8E-09	8,7E-07	bta-miR-423-5p	-0,7	6,6E-06	2,4E-03	bta-miR-130a	-3,8	6,4E-06	2,3E-03
bta-miR-486	-2,9	1,7E-07	2,0E-05	bta-miR-34a	6,6	2,2E-04	1,6E-02	bta-miR-181b	-8,4	2,0E-04	3,7E-02
bta-let-7b	-5,4	1,9E-07	2,0E-05	bta-miR-146b	2,8	3,3E-04	1,6E-02	bta-miR-2457	7,6	2,9E-03	2,8E-01
bta-miR-665	-3,4	5,5E-05	4,3E-03	Novel:X_44465	3,1	3,5E-04	1,6E-02	bta-miR-769	0,9	3,1E-03	2,8E-01
bta-let-7a-5p	-2,1	9,6E-05	5,5E-03	bta-miR-221	3,7	3,6E-04	1,6E-02	bta-miR-296-5p	5,0	9,7E-03	4,5E-01
bta-miR-451	-8,1	1,3E-04	5,5E-03	Novel:X_44461	3,1	3,6E-04	1,6E-02	Novel:eca-miR-211	-2,9	1,0E-02	4,5E-01
bta-let-7e	-3,9	1,3E-04	5,5E-03	Novel:X_44459	3,1	3,8E-04	1,6E-02	Novel:8_41340	-6,6	1,0E-02	4,5E-01
Novel:eca-let-7a	-5,1	1,4E-04	5,5E-03	Novel:X_44457	3,1	3,8E-04	1,6E-02	bta-miR-204	-2,9	1,2E-02	4,5E-01
bta-let-7g	-3,2	2,7E-04	9,3E-03	Novel:X_44433	3,1	3,9E-04	1,6E-02	bta-let-7b	-2,7	1,4E-02	4,5E-01
bta-miR-200a	-6,6	8,2E-04	2,5E-02	bta-miR-10a	-3,3	4,4E-04	1,6E-02	bta-miR-221	-3,1	1,5E-02	4,5E-01
bta-miR-181a	-2,2	8,7E-04	2,5E-02	bta-miR-486	-2,1	6,1E-04	2,0E-02	Novel:2_20026	-6,1	1,7E-02	4,5E-01
bta-miR-182	-1,9	1,7E-03	4,1E-02	bta-let-7e	-2,4	1,3E-03	3,9E-02	Novel:13_6477	-6,1	1,7E-02	4,5E-01
bta-miR-296-5p	7,6	1,7E-03	4,1E-02	Novel:X_44463	6,3	1,6E-03	4,3E-02	Novel:eca-miR-367	-6,0	1,7E-02	4,5E-01
bta-miR-1246	1,8	2,0E-03	4,4E-02	bta-miR-6526	3,1	1,6E-03	4,3E-02	bta-miR-32	-6,0	1,7E-02	4,5E-01
bta-miR-375	-2,7	2,3E-03	4,8E-02	Novel:6_37889	8,4	1,9E-03	4,6E-02	Novel:eca-miR-628a	-5,8	2,2E-02	5,3E-01
Novel:X_44463	6,2	3,5E-03	6,7E-02	Novel:5_36094	7,5	2,2E-03	4,9E-02	bta-miR-628	-5,8	2,4E-02	5,3E-01
bta-miR-197	6,8	5,2E-03	9,5E-02	bta-miR-1247-5p	7,4	2,5E-03	4,9E-02	bta-miR-363	-5,5	2,8E-02	5,3E-01
bta-miR-10b	1,1	5,8E-03	1,0E-01	Novel:eca-miR-450b-5p	5,9	2,6E-03	4,9E-02	Novel:23_24577	-5,2	2,9E-02	5,3E-01
Novel:eca-miR-181a	-2,2	6,6E-03	1,0E-01	bta-miR-2316	7,4	2,6E-03	4,9E-02	Novel:X_45194	-5,5	2,9E-02	5,3E-01
bta-miR-449a	3,1	6,6E-03	1,0E-01	bta-miR-450b	5,9	2,7E-03	4,9E-02	Novel:23_23290	-5,2	2,9E-02	5,3E-01
bta-miR-148a	1,1	6,7E-03	1,0E-01	bta-miR-320a	2,9	3,1E-03	5,4E-02	bta-miR-665	-2,0	4,0E-02	7,0E-01
bta-miR-2457	7,1	7,6E-03	1,1E-01	bta-miR-6123	7,3	3,3E-03	5,4E-02	bta-miR-6526	-1,1	4,4E-02	7,1E-01
Novel:27_28687	5,5	8,2E-03	1,1E-01	bta-miR-449a	3,4	4,5E-03	7,1E-02	bta-miR-2285k	-1,7	4,4E-02	7,1E-01

bta-miR-199a-3p	-4,4	1,1E-02	1,3E-01	bta-miR-335	7,1	5,8E-03	8,8E-02	bta-miR-296-3p	0,9	5,7E-02	8,5E-01
bta-miR-1291	-5,9	1,1E-02	1,3E-01	bta-miR-200a	-5,4	6,0E-03	8,8E-02	bta-miR-34a	-2,4	5,9E-02	8,5E-01
Novel:eca-miR-200a	-4,2	1,1E-02	1,3E-01	Novel:8_41340	6,5	6,3E-03	8,9E-02	bta-miR-320a	-2,1	6,0E-02	8,5E-01
bta-miR-143	-1,6	1,2E-02	1,4E-01	bta-miR-130a	4,4	7,3E-03	9,9E-02	bta-miR-23b-3p	-1,9	6,6E-02	9,0E-01
bta-miR-6526	1,9	2,1E-02	2,1E-01	bta-miR-182	-1,3	9,6E-03	1,3E-01	bta-miR-155	-1,6	7,1E-02	9,3E-01
bta-miR-127	-2,0	2,1E-02	2,1E-01	Novel:17_14163	6,5	1,2E-02	1,6E-01	bta-miR-23a	-2,4	7,8E-02	9,9E-01
bta-miR-450a	5,2	2,1E-02	2,1E-01	Novel:eca-let-7a	-2,6	1,4E-02	1,7E-01	bta-miR-331-3p	1,8	8,6E-02	9,9E-01
bta-miR-22-5p	-4,9	2,2E-02	2,1E-01	Novel:X_44441	4,8	1,4E-02	1,7E-01	bta-miR-219	2,2	9,1E-02	9,9E-01
bta-miR-146b	2,1	2,2E-02	2,1E-01	Novel:2_20026	6,1	1,5E-02	1,7E-01	bta-let-7g	-1,5	1,0E-01	9,9E-01
bta-miR-141	-3,1	2,3E-02	2,2E-01	Novel:eca-miR-34a	3,1	1,6E-02	1,7E-01	bta-miR-2344	1,4	1,1E-01	9,9E-01
bta-miR-744	-1,3	2,6E-02	2,3E-01	Novel:13_6477	6,1	1,6E-02	1,7E-01	bta-miR-2285g	-1,4	1,1E-01	9,9E-01
Novel:6_36682	-1,5	2,7E-02	2,3E-01	Novel:eca-miR-367	6,0	1,7E-02	1,7E-01	bta-miR-1247-5p	-2,9	1,2E-01	9,9E-01
bta-miR-450b	4,3	2,7E-02	2,3E-01	bta-let-7b	-2,5	1,7E-02	1,7E-01	bta-miR-1291	-3,2	1,4E-01	9,9E-01
Novel:eca-miR-450b-5p	4,3	2,8E-02	2,3E-01	bta-miR-32	6,0	1,7E-02	1,7E-01	bta-miR-2440	1,9	1,4E-01	9,9E-01
bta-let-7f	-1,0	3,0E-02	2,4E-01	Novel:eca-miR-1301	5,7	2,0E-02	1,9E-01	bta-miR-127	-0,6	1,5E-01	9,9E-01
bta-miR-23b-3p	-2,2	4,0E-02	3,2E-01	bta-miR-4449	6,0	2,1E-02	2,0E-01	bta-miR-2284y	-1,1	1,6E-01	9,9E-01
bta-miR-411a	-1,4	4,4E-02	3,4E-01	bta-miR-219	-3,1	2,4E-02	2,2E-01	bta-miR-181a	-1,2	1,7E-01	9,9E-01
bta-miR-2320-3p	4,7	4,4E-02	3,4E-01	bta-miR-375	-2,7	2,5E-02	2,2E-01	bta-miR-184	-0,3	1,8E-01	9,9E-01
Novel:eca-miR-411	-1,7	5,0E-02	3,4E-01	bta-miR-363	5,6	3,0E-02	2,5E-01	bta-miR-423-3p	0,7	1,8E-01	9,9E-01
Novel:eca-miR-199a-3p	-3,6	5,1E-02	3,4E-01	Novel:X_45194	5,6	3,0E-02	2,5E-01	bta-miR-450b	-1,5	1,9E-01	9,9E-01
bta-miR-660	0,8	5,1E-02	3,4E-01	bta-miR-148a	1,1	3,1E-02	2,5E-01	bta-miR-128	0,5	1,9E-01	9,9E-01
bta-miR-142-5p	-3,5	5,4E-02	3,4E-01	bta-miR-1271	1,8	3,1E-02	2,5E-01	Novel:eca-miR-450b-5p	-1,5	1,9E-01	9,9E-01
Novel:eca-miR-450a	4,3	5,4E-02	3,4E-01	Novel:23_24577	5,2	3,4E-02	2,7E-01	bta-miR-193b	3,2	1,9E-01	9,9E-01
Novel:19_17483	-3,5	5,4E-02	3,4E-01	Novel:23_23290	5,2	3,4E-02	2,7E-01	Novel:18_16297	2,8	2,0E-01	9,9E-01
bta-miR-493	-4,0	5,5E-02	3,4E-01	bta-miR-2478	-2,3	3,6E-02	2,7E-01	bta-miR-34c	-0,4	2,0E-01	9,9E-01
Novel:eca-miR-493b	-4,0	5,5E-02	3,4E-01	bta-miR-205	2,3	4,0E-02	2,9E-01	Novel:eca-let-7a	-2,5	2,0E-01	9,9E-01
bta-miR-181c	-3,4	5,6E-02	3,4E-01	bta-miR-204	1,7	4,1E-02	2,9E-01	bta-miR-2400	-3,1	2,1E-01	9,9E-01
Novel:X_44451	4,1	6,0E-02	3,4E-01	Novel:eca-miR-205	2,3	4,1E-02	2,9E-01	bta-let-7e	-1,5	2,1E-01	9,9E-01
Novel:X_44461	2,0	6,1E-02	3,4E-01	Novel:eca-miR-211	1,7	4,1E-02	2,9E-01	Novel:12_5433	1,2	2,1E-01	9,9E-01

Novel:X_44459	2,0	6,1E-02	3,4E-01	Novel:oar-miR-541-5p	-4,6	4,2E-02	2,9E-01	bta-miR-140	1,1	2,1E-01	9,9E-01
Novel:X_44441	4,5	6,1E-02	3,4E-01	Novel:X_44451	4,8	5,1E-02	3,4E-01	bta-miR-2284ab	1,2	2,1E-01	9,9E-01
Novel:X_44465	2,0	6,1E-02	3,4E-01	bta-let-7g	-1,5	5,3E-02	3,4E-01	Novel:eca-miR-181a	-1,2	2,1E-01	9,9E-01
Novel:X_44457	2,0	6,1E-02	3,4E-01	Novel:eca-miR-146b-5p	3,8	5,3E-02	3,4E-01	bta-miR-532	0,4	2,2E-01	9,9E-01
Novel:X_44433	2,0	6,2E-02	3,4E-01	bta-miR-2427	-3,7	5,9E-02	3,8E-01	Novel:chi-miR-151-3p	0,4	2,2E-01	9,9E-01
bta-miR-1260b	-1,7	6,4E-02	3,5E-01	bta-let-7a-5p	-1,7	6,0E-02	3,8E-01	bta-miR-302a	-2,6	2,2E-01	9,9E-01
bta-miR-92b	-0,8	6,9E-02	3,7E-01	bta-miR-2285f	2,0	6,1E-02	3,8E-01	bta-miR-2340	-2,7	2,2E-01	9,9E-01
Novel:16_12344	1,6	7,5E-02	3,9E-01	bta-miR-1246	2,1	6,8E-02	4,1E-01	bta-miR-19a	1,4	2,2E-01	9,9E-01
bta-miR-2887	2,1	7,7E-02	3,9E-01	Novel:27_28687	4,5	6,8E-02	4,1E-01	bta-miR-378b	1,0	2,3E-01	9,9E-01
bta-miR-504	-2,4	7,9E-02	4,0E-01	bta-miR-25	-0,6	7,0E-02	4,1E-01	bta-miR-2478	1,0	2,4E-01	9,9E-01
Novel:eca-miR-1839	-3,5	8,3E-02	4,1E-01	bta-miR-2285c	-4,2	7,1E-02	4,1E-01	bta-miR-2389	2,7	2,4E-01	9,9E-01
bta-miR-1839	-3,5	8,5E-02	4,1E-01	bta-miR-192	0,4	7,7E-02	4,4E-01	Novel:29_29980	-1,2	2,4E-01	9,9E-01
bta-miR-34a	4,1	9,1E-02	4,3E-01	Novel:eca-miR-192	0,4	7,7E-02	4,4E-01	Novel:28_29427	-1,2	2,4E-01	9,9E-01
bta-let-7d	-1,6	9,3E-02	4,3E-01	bta-miR-2397-3p	-4,1	8,0E-02	4,4E-01	bta-miR-374a	-2,2	2,4E-01	9,9E-01
Novel:eca-miR-34a	2,3	9,5E-02	4,3E-01	bta-miR-423-3p	-1,2	8,2E-02	4,5E-01	bta-miR-2447	2,4	2,4E-01	9,9E-01
bta-miR-154c	-3,4	9,5E-02	4,3E-01	Novel:4_34592	2,9	8,4E-02	4,6E-01	bta-miR-1271	-1,0	2,5E-01	9,9E-01
Novel:oar-miR-3958-3p	-3,4	9,6E-02	4,3E-01	Novel:eca-miR-200a	-2,1	8,6E-02	4,6E-01	Novel:28_29233	-2,2	2,5E-01	9,9E-01
Novel:7_40910	4,2	1,0E-01	4,4E-01	bta-miR-196b	-3,6	8,9E-02	4,6E-01	Novel:24_26327	-2,5	2,5E-01	9,9E-01
bta-miR-183	-2,1	1,0E-01	4,5E-01	Novel:eca-miR-196b	-3,6	8,9E-02	4,6E-01	bta-miR-30c	0,3	2,5E-01	9,9E-01
bta-miR-2478	-1,4	1,1E-01	4,6E-01	Novel:6_36677	-3,5	9,3E-02	4,6E-01	Novel:eca-miR-205	-1,2	2,5E-01	9,9E-01
bta-miR-29a	-2,1	1,1E-01	4,6E-01	bta-miR-677	-1,5	9,3E-02	4,6E-01	bta-miR-486	-0,6	2,5E-01	9,9E-01
bta-miR-1343-3p	-1,4	1,1E-01	4,7E-01	bta-miR-2285t	-3,5	9,4E-02	4,6E-01	bta-miR-205	-1,2	2,5E-01	9,9E-01
Novel:eca-miR-146b-5p	2,9	1,2E-01	5,0E-01	Novel:18_16297	-3,4	9,5E-02	4,6E-01	bta-miR-26b	0,4	2,6E-01	9,9E-01
bta-miR-361	-1,5	1,2E-01	5,0E-01	bta-miR-1260b	-2,2	9,5E-02	4,6E-01	bta-let-7c	-2,1	2,7E-01	9,9E-01
bta-miR-26a	-0,6	1,4E-01	5,6E-01	bta-miR-2447	-3,4	9,5E-02	4,6E-01	bta-miR-200c	2,5	2,8E-01	9,9E-01
Novel:eca-miR-26a	-0,6	1,4E-01	5,7E-01	bta-miR-199a-3p	-2,3	9,7E-02	4,6E-01	bta-miR-335	-2,2	2,8E-01	9,9E-01
bta-miR-423-3p	-0,6	1,4E-01	5,7E-01	Novel:6_37391	1,6	9,9E-02	4,6E-01	bta-miR-20a	0,8	2,8E-01	9,9E-01
bta-miR-155	-1,1	1,5E-01	5,7E-01	bta-miR-665	-1,3	9,9E-02	4,6E-01	bta-miR-411a	-0,6	2,8E-01	9,9E-01
bta-miR-423-5p	-0,6	1,5E-01	5,7E-01	bta-miR-142-5p	-3,1	1,0E-01	4,6E-01	bta-miR-1839	-2,1	2,8E-01	9,9E-01

bta-miR-2314	2,5	1,5E-01	5,8E-01	Novel:19_17483	-3,1	1,0E-01	4,6E-01	Novel:eca-miR-1839	-2,1	2,9E-01	9,9E-01
Novel:7_39799	-2,9	1,5E-01	5,8E-01	Novel:eca-miR-199a-3p	-2,7	1,1E-01	4,7E-01	Novel:X_45264	-1,6	2,9E-01	9,9E-01
bta-miR-3432a	-1,1	1,6E-01	5,8E-01	Novel:eca-miR-143	-1,4	1,1E-01	4,7E-01	bta-miR-141	-1,5	2,9E-01	9,9E-01
bta-miR-2285aa	2,1	1,6E-01	5,8E-01	Novel:eca-miR-369-5p	-3,0	1,1E-01	4,8E-01	bta-miR-4449	-2,2	2,9E-01	9,9E-01
bta-miR-27a-3p	-1,3	1,6E-01	5,8E-01	bta-miR-369-5p	-3,0	1,1E-01	4,8E-01	Novel:X_44433	-1,1	2,9E-01	9,9E-01
Novel:eca-miR-143	-1,4	1,6E-01	5,8E-01	Novel:6_39307	1,3	1,1E-01	4,8E-01	bta-miR-1307	1,0	2,9E-01	9,9E-01
bta-miR-2885	2,9	1,7E-01	5,9E-01	bta-let-7d	-2,3	1,1E-01	4,8E-01	Novel:X_44459	-1,1	2,9E-01	9,9E-01
bta-miR-7857	1,5	1,7E-01	6,0E-01	Novel:29_29980	2,1	1,2E-01	5,0E-01	Novel:X_44457	-1,1	2,9E-01	9,9E-01
bta-miR-27b	-0,6	1,8E-01	6,1E-01	Novel:28_29427	2,1	1,2E-01	5,0E-01	Novel:X_44461	-1,1	3,0E-01	9,9E-01
bta-miR-98	-2,6	1,8E-01	6,1E-01	bta-miR-21-5p	0,7	1,3E-01	5,0E-01	Novel:X_44465	-1,1	3,0E-01	9,9E-01
bta-miR-222	1,0	1,8E-01	6,1E-01	Novel:eca-miR-21	0,7	1,3E-01	5,0E-01	bta-miR-378d	2,0	3,0E-01	9,9E-01
Novel:eca-miR-31	-1,6	1,9E-01	6,1E-01	bta-miR-22-3p	0,2	1,3E-01	5,0E-01	Novel:chi-miR-3432-5p	-0,9	3,0E-01	9,9E-01
bta-miR-31	-1,6	1,9E-01	6,1E-01	bta-miR-2320-3p	3,5	1,3E-01	5,0E-01	bta-miR-2318	1,2	3,0E-01	9,9E-01
bta-miR-302a	-2,7	1,9E-01	6,1E-01	Novel:21_21851	-2,6	1,3E-01	5,0E-01	Novel:15_11311	-1,9	3,0E-01	9,9E-01
Novel:chi-miR-3432-5p	-1,3	1,9E-01	6,1E-01	bta-miR-181c	-2,1	1,4E-01	5,0E-01	bta-miR-183	-1,3	3,1E-01	9,9E-01
bta-miR-2285k	-1,4	1,9E-01	6,1E-01	Novel:23_24075	-3,1	1,4E-01	5,0E-01	bta-miR-27a-3p	-0,8	3,1E-01	9,9E-01
bta-miR-2427	-2,5	1,9E-01	6,2E-01	bta-miR-29c	-3,0	1,4E-01	5,0E-01	Novel:23_24587	1,0	3,1E-01	9,9E-01
bta-miR-2285q	2,0	2,0E-01	6,2E-01	bta-miR-22-5p	-2,7	1,4E-01	5,0E-01	Novel:23_23280	1,0	3,1E-01	9,9E-01
bta-miR-342	-1,8	2,0E-01	6,2E-01	Novel:X_45264	2,4	1,4E-01	5,0E-01	bta-miR-146b	-0,7	3,2E-01	9,9E-01
Novel:6_38564	0,7	2,0E-01	6,2E-01	bta-miR-1306	-3,0	1,4E-01	5,0E-01	Novel:eca-miR-200a	-1,9	3,2E-01	9,9E-01
bta-miR-677	-1,1	2,1E-01	6,4E-01	bta-miR-1291	-2,7	1,4E-01	5,0E-01	Novel:eca-miR-101	0,9	3,3E-01	9,9E-01
Novel:15_11027	-1,9	2,1E-01	6,4E-01	bta-miR-140	-1,0	1,4E-01	5,0E-01	bta-miR-199a-3p	-1,9	3,3E-01	9,9E-01
Novel:X_45065	0,6	2,2E-01	6,5E-01	Novel:8_42756	1,4	1,4E-01	5,1E-01	bta-miR-1248	-2,0	3,3E-01	9,9E-01
Novel:oar-miR-323c	-2,3	2,2E-01	6,5E-01	Novel:6_36682	-1,3	1,5E-01	5,1E-01	Novel:6_37889	-1,5	3,3E-01	9,9E-01
bta-miR-497	-2,6	2,2E-01	6,5E-01	bta-miR-744	-1,4	1,5E-01	5,2E-01	bta-miR-338	-1,1	3,4E-01	9,9E-01
bta-miR-186	-0,5	2,2E-01	6,5E-01	bta-miR-100	2,5	1,6E-01	5,4E-01	bta-miR-2316	-1,6	3,5E-01	9,9E-01
Novel:eca-miR-186	-0,5	2,2E-01	6,5E-01	Novel:eca-miR-100	2,5	1,6E-01	5,4E-01	bta-miR-6119-3p	-1,8	3,6E-01	9,9E-01
bta-miR-2336	1,3	2,3E-01	6,5E-01	Novel:20_20852	2,1	1,6E-01	5,4E-01	bta-miR-410	-1,1	3,6E-01	9,9E-01
bta-miR-219	-1,0	2,3E-01	6,5E-01	bta-miR-2285q	2,1	1,6E-01	5,4E-01	bta-miR-27b	-0,8	3,7E-01	9,9E-01

bta-miR-6119-5p	0,6	2,4E-01	6,6E-01	bta-miR-296-3p	-1,1	1,6E-01	5,4E-01	bta-miR-340	1,6	3,7E-01	9,9E-01
Novel:8_42756	0,9	2,4E-01	6,7E-01	Novel:eca-miR-22	0,2	1,6E-01	5,4E-01	bta-miR-10a	-0,5	3,7E-01	9,9E-01
bta-miR-92a	-0,4	2,5E-01	6,7E-01	bta-miR-2885	3,1	1,7E-01	5,5E-01	bta-miR-99b	-0,8	3,7E-01	9,9E-01
Novel:3_32033	1,3	2,5E-01	6,7E-01	bta-miR-2284y	1,4	1,7E-01	5,5E-01	bta-miR-18a	1,6	3,8E-01	9,9E-01
bta-miR-21-5p	0,5	2,5E-01	6,7E-01	Novel:7_39799	-2,3	1,8E-01	5,6E-01	Novel:15_11027	-1,2	3,8E-01	9,9E-01
Novel:eca-miR-21	0,4	2,5E-01	6,7E-01	Novel:28_29233	2,3	1,8E-01	5,8E-01	Novel:6_37391	-0,7	3,8E-01	9,9E-01
Novel:21_21851	-2,1	2,6E-01	6,9E-01	bta-miR-361	-1,0	1,9E-01	6,1E-01	bta-miR-182	-0,5	3,8E-01	9,9E-01
Novel:eca-miR-192	0,5	2,7E-01	6,9E-01	bta-miR-1307	-0,9	1,9E-01	6,1E-01	bta-miR-421	-0,6	3,9E-01	9,9E-01
bta-miR-151-3p	-0,5	2,7E-01	6,9E-01	bta-miR-141	-1,5	2,0E-01	6,1E-01	bta-miR-2435	-1,6	3,9E-01	9,9E-01
bta-miR-30f	-2,0	2,7E-01	6,9E-01	bta-miR-92a	-1,0	2,0E-01	6,1E-01	bta-miR-2285b	0,8	4,0E-01	9,9E-01
bta-miR-192	0,4	2,7E-01	6,9E-01	bta-miR-2887	2,1	2,0E-01	6,1E-01	bta-miR-21-3p	-0,8	4,0E-01	9,9E-01
bta-miR-410	-1,4	2,7E-01	6,9E-01	bta-miR-1343-3p	-1,5	2,1E-01	6,3E-01	bta-miR-2285f	-0,7	4,1E-01	9,9E-01
bta-miR-9-5p	0,7	2,7E-01	6,9E-01	Novel:chi-miR-2284d	1,7	2,1E-01	6,3E-01	bta-miR-493	-1,8	4,2E-01	9,9E-01
bta-miR-378d	2,0	2,8E-01	6,9E-01	Novel:12_5433	-1,3	2,2E-01	6,3E-01	Novel:eca-miR-493b	-1,8	4,2E-01	9,9E-01
Novel:eca-miR-148a	0,7	2,8E-01	7,0E-01	Novel:oar-miR-379-3p	-2,3	2,2E-01	6,3E-01	Novel:7_40910	1,5	4,2E-01	9,9E-01
bta-miR-148b	0,7	2,9E-01	7,1E-01	bta-miR-127	-1,2	2,2E-01	6,3E-01	bta-miR-101	0,5	4,2E-01	9,9E-01
Novel:4_34592	2,2	2,9E-01	7,1E-01	Novel:oar-miR-3958-3p	-1,8	2,2E-01	6,3E-01	Novel:27_28687	1,3	4,2E-01	9,9E-01
bta-miR-320a	0,6	2,9E-01	7,1E-01	bta-miR-154c	-1,8	2,2E-01	6,3E-01	bta-let-7a-5p	-0,3	4,2E-01	9,9E-01
bta-miR-2285f	1,2	3,0E-01	7,2E-01	bta-miR-378b	-0,9	2,2E-01	6,3E-01	bta-miR-93	0,2	4,3E-01	9,9E-01
bta-miR-133a	0,8	3,2E-01	7,5E-01	bta-miR-143	-1,2	2,2E-01	6,3E-01	Novel:16_12344	0,7	4,3E-01	9,9E-01
Novel:6_39307	0,9	3,2E-01	7,5E-01	bta-miR-660	0,9	2,3E-01	6,3E-01	Novel:16_12359	-1,6	4,3E-01	9,9E-01
bta-miR-129-5p	2,3	3,2E-01	7,5E-01	Novel:eca-miR-493b	-2,2	2,3E-01	6,3E-01	bta-miR-6120-3p	1,2	4,3E-01	9,9E-01
bta-miR-129	2,3	3,2E-01	7,5E-01	Novel:oar-miR-323c	-2,0	2,3E-01	6,3E-01	Novel:eca-miR-34a	-0,7	4,3E-01	9,9E-01
Novel:chi-miR-151-3p	-0,4	3,4E-01	7,8E-01	bta-miR-493	-2,2	2,3E-01	6,3E-01	bta-miR-222	0,6	4,3E-01	9,9E-01
bta-miR-484	-1,0	3,4E-01	7,8E-01	bta-miR-16a	1,1	2,4E-01	6,3E-01	Novel:5_35327	-1,5	4,4E-01	9,9E-01
bta-miR-2344	1,4	3,4E-01	7,8E-01	Novel:eca-miR-15b	1,1	2,4E-01	6,3E-01	bta-miR-125a	-0,3	4,4E-01	9,9E-01
bta-miR-302b	-2,1	3,5E-01	7,9E-01	bta-miR-2284z	0,8	2,4E-01	6,4E-01	bta-miR-194	0,6	4,5E-01	9,9E-01
bta-miR-106b	-1,4	3,5E-01	7,9E-01	bta-miR-2340	2,6	2,4E-01	6,4E-01	bta-miR-151-3p	0,3	4,5E-01	9,9E-01
Novel:16_12359	-1,9	3,6E-01	7,9E-01	bta-miR-26a	-0,3	2,4E-01	6,4E-01	Novel:eca-miR-186	0,2	4,6E-01	9,9E-01
bta-miR-17-3p	2,1	3,7E-01	8,2E-01	bta-miR-374a	1,7	2,5E-01	6,4E-01	Novel:8_42756	-0,4	4,6E-01	9,9E-01

Novel:6_37391	0,8	3,8E-01	8,3E-01	Novel:7_40910	2,8	2,5E-01	6,4E-01	Novel:oar-miR-3958-3p	-1,4	4,6E-01	9,9E-01
bta-miR-1271	0,8	3,9E-01	8,4E-01	bta-miR-338	1,5	2,6E-01	6,7E-01	bta-miR-154c	-1,4	4,6E-01	9,9E-01
Novel:chi-miR-374a-3p	-1,8	3,9E-01	8,4E-01	bta-miR-2399-5p	-2,1	2,7E-01	6,8E-01	bta-miR-2285aa	0,7	4,6E-01	9,9E-01
bta-miR-2284ab	0,6	3,9E-01	8,4E-01	bta-miR-148b	0,7	2,7E-01	6,8E-01	bta-miR-2904	-0,7	4,6E-01	9,9E-01
Novel:20_20852	1,5	4,0E-01	8,5E-01	bta-miR-129-5p	2,0	2,7E-01	6,8E-01	bta-miR-2314	0,8	4,7E-01	9,9E-01
Novel:6_37493	0,6	4,0E-01	8,5E-01	bta-miR-129	2,0	2,7E-01	6,8E-01	Novel:6_36914	-1,2	4,7E-01	9,9E-01
Novel:eca-miR-1468	-0,4	4,0E-01	8,5E-01	Novel:eca-miR-148a	0,7	2,8E-01	6,9E-01	bta-miR-186	0,2	4,7E-01	9,9E-01
bta-miR-1468	-0,4	4,1E-01	8,5E-01	bta-miR-6120-3p	-1,5	2,9E-01	7,1E-01	bta-miR-2320-3p	1,4	4,8E-01	9,9E-01
bta-miR-204	-1,3	4,1E-01	8,5E-01	bta-miR-331-3p	-1,4	2,9E-01	7,1E-01	Novel:eca-miR-132	1,5	4,8E-01	9,9E-01
Novel:eca-miR-211	-1,3	4,1E-01	8,5E-01	Novel:eca-miR-129a-5p	1,9	2,9E-01	7,1E-01	Novel:14_9885	-1,2	4,8E-01	9,9E-01
bta-miR-302d	-1,6	4,2E-01	8,5E-01	bta-miR-23a	1,6	3,0E-01	7,1E-01	Novel:13_7227	-1,5	4,8E-01	9,9E-01
Novel:eca-miR-369-5p	-1,7	4,3E-01	8,5E-01	bta-let-7f	-0,7	3,0E-01	7,1E-01	bta-miR-17-5p	0,6	4,8E-01	9,9E-01
bta-miR-369-5p	-1,7	4,3E-01	8,5E-01	bta-miR-2484	-1,6	3,0E-01	7,1E-01	bta-miR-484	-0,6	4,8E-01	9,9E-01
Novel:eca-miR-129a-5p	1,7	4,3E-01	8,5E-01	bta-miR-24-3p	1,8	3,0E-01	7,1E-01	Novel:eca-miR-411	-0,7	4,9E-01	9,9E-01
bta-miR-30b-5p	-0,8	4,3E-01	8,5E-01	Novel:16_12344	1,0	3,0E-01	7,1E-01	bta-miR-3432a	-0,7	4,9E-01	9,9E-01
Novel:eca-miR-30c	-0,8	4,3E-01	8,5E-01	Novel:eca-miR-31	-1,1	3,0E-01	7,1E-01	Novel:eca-miR-1468	0,3	4,9E-01	9,9E-01
Novel:eca-miR-221	0,7	4,3E-01	8,5E-01	bta-miR-31	-1,1	3,1E-01	7,1E-01	bta-miR-1468	0,3	4,9E-01	9,9E-01
bta-miR-205	0,9	4,4E-01	8,5E-01	bta-miR-2314	1,8	3,1E-01	7,3E-01	bta-miR-425-3p	-1,5	5,0E-01	9,9E-01
Novel:eca-miR-205	0,9	4,4E-01	8,5E-01	bta-miR-7	2,2	3,2E-01	7,3E-01	bta-miR-378c	0,6	5,0E-01	9,9E-01
bta-miR-25	-0,3	4,4E-01	8,5E-01	bta-miR-29a	-1,2	3,2E-01	7,4E-01	Novel:eca-miR-194	0,5	5,1E-01	9,9E-01
bta-miR-424-3p	-1,2	4,4E-01	8,5E-01	bta-miR-200c	-2,0	3,4E-01	7,7E-01	bta-miR-365-3p	-1,5	5,1E-01	9,9E-01
Novel:chi-miR-2284d	1,2	4,4E-01	8,5E-01	bta-miR-10b	1,3	3,5E-01	7,8E-01	Novel:5_36094	-1,2	5,1E-01	9,9E-01
bta-miR-210	0,7	4,5E-01	8,5E-01	bta-miR-342	-1,0	3,5E-01	7,8E-01	bta-miR-2285e	-1,3	5,1E-01	9,9E-01
bta-miR-2284v	1,5	4,5E-01	8,5E-01	bta-miR-2404	-1,7	3,6E-01	7,8E-01	Novel:17_14702	1,6	5,1E-01	9,9E-01
bta-miR-340	1,7	4,6E-01	8,6E-01	Novel:eca-miR-1839	-1,3	3,6E-01	7,8E-01	bta-miR-17-3p	1,2	5,2E-01	9,9E-01
bta-miR-151-5p	-0,4	4,6E-01	8,6E-01	Novel:eca-miR-181a	-0,8	3,6E-01	7,8E-01	bta-miR-28	-0,4	5,2E-01	9,9E-01
bta-miR-19b	0,6	4,6E-01	8,6E-01	bta-miR-1839	-1,3	3,6E-01	7,8E-01	bta-miR-302d	-1,2	5,2E-01	9,9E-01
Novel:19_16745	0,8	4,7E-01	8,7E-01	Novel:eca-miR-411	-0,9	3,6E-01	7,8E-01	bta-miR-98	-1,3	5,3E-01	9,9E-01

bta-miR-15a	0,8	4,7E-01	8,7E-01	Novel:chi-miR-374a-3p	-1,6	3,6E-01	7,8E-01	bta-miR-411c-5p	1,2	5,3E-01	9,9E-01
Novel:16_11586	1,5	4,8E-01	8,7E-01	bta-miR-2285aa	1,7	3,6E-01	7,8E-01	bta-miR-200a	-1,3	5,4E-01	9,9E-01
bta-miR-2484	-1,0	4,8E-01	8,7E-01	bta-miR-2336	1,1	3,6E-01	7,8E-01	bta-miR-6524	-1,2	5,4E-01	9,9E-01
bta-miR-16b	0,3	4,8E-01	8,8E-01	bta-miR-7857	1,0	3,6E-01	7,8E-01	Novel:eca-miR-15b	-0,6	5,5E-01	9,9E-01
bta-miR-24-3p	1,3	4,9E-01	8,8E-01	bta-miR-133a	0,7	3,7E-01	7,8E-01	bta-miR-424-5p	1,2	5,6E-01	9,9E-01
bta-miR-93	-0,4	4,9E-01	8,8E-01	Novel:eca-miR-7	1,9	3,7E-01	7,8E-01	bta-miR-16a	-0,6	5,6E-01	9,9E-01
bta-miR-23a	-1,0	5,0E-01	8,8E-01	bta-miR-2904	0,9	3,8E-01	7,8E-01	bta-miR-2284z	-0,6	5,6E-01	9,9E-01
bta-miR-20a	0,7	5,1E-01	8,9E-01	bta-miR-19a	-1,3	3,8E-01	7,8E-01	bta-miR-345-3p	1,2	5,6E-01	9,9E-01
bta-miR-2285g	-0,8	5,1E-01	8,9E-01	bta-miR-504	-1,6	3,8E-01	7,9E-01	bta-miR-342	-0,7	5,7E-01	9,9E-01
bta-miR-15b	0,6	5,2E-01	9,0E-01	bta-miR-431	-1,9	3,9E-01	8,0E-01	bta-miR-181c	-1,1	5,7E-01	9,9E-01
bta-miR-1248	-1,2	5,2E-01	9,0E-01	Novel:X_44449	1,7	3,9E-01	8,0E-01	Novel:15_10278	1,1	5,7E-01	9,9E-01
bta-miR-2404	-1,2	5,2E-01	9,0E-01	bta-miR-424-3p	-1,2	3,9E-01	8,0E-01	bta-miR-361	-0,5	5,7E-01	9,9E-01
bta-miR-421	-0,6	5,3E-01	9,0E-01	bta-miR-374b	-2,0	3,9E-01	8,0E-01	Novel:eca-miR-221	0,5	5,7E-01	9,9E-01
bta-miR-381	-0,5	5,3E-01	9,0E-01	bta-miR-654	-1,8	4,0E-01	8,0E-01	Novel:eca-miR-105	-1,2	5,8E-01	9,9E-01
Novel:eca-miR-9040	1,3	5,3E-01	9,0E-01	Novel:eca-miR-1468	-0,7	4,0E-01	8,0E-01	bta-miR-105b	-1,2	5,8E-01	9,9E-01
bta-miR-652	-1,2	5,4E-01	9,1E-01	bta-miR-1468	-0,7	4,0E-01	8,0E-01	bta-miR-369-5p	1,3	5,8E-01	9,9E-01
bta-miR-296-3p	-0,2	5,5E-01	9,2E-01	bta-miR-151-3p	-0,7	4,0E-01	8,0E-01	Novel:eca-miR-369-5p	1,3	5,8E-01	9,9E-01
bta-miR-2284aa	1,3	5,5E-01	9,2E-01	bta-miR-15a	0,9	4,1E-01	8,1E-01	bta-miR-106b	-0,8	5,9E-01	9,9E-01
Novel:21_21202	1,1	5,5E-01	9,2E-01	bta-miR-2440	-1,2	4,2E-01	8,2E-01	bta-miR-2346	0,4	5,9E-01	9,9E-01
bta-miR-149-5p	-0,6	5,6E-01	9,3E-01	Novel:X_45065	0,7	4,2E-01	8,2E-01	bta-miR-6529a	0,2	6,0E-01	9,9E-01
bta-miR-17-5p	0,4	5,7E-01	9,3E-01	Novel:chi-miR-151-3p	-0,7	4,3E-01	8,3E-01	Novel:21_21202	0,8	6,0E-01	9,9E-01
bta-miR-2440	0,6	5,7E-01	9,4E-01	bta-miR-6119-5p	0,7	4,4E-01	8,3E-01	bta-miR-7857	0,5	6,0E-01	9,9E-01
bta-miR-2447	-1,0	5,8E-01	9,4E-01	Novel:21_21549	-1,4	4,4E-01	8,3E-01	bta-miR-677	0,5	6,0E-01	9,9E-01
bta-let-7i	-0,3	5,8E-01	9,4E-01	bta-miR-628	1,5	4,5E-01	8,3E-01	bta-let-7i	-0,3	6,0E-01	9,9E-01
bta-miR-125a	-0,3	5,9E-01	9,4E-01	bta-miR-28	0,7	4,5E-01	8,3E-01	bta-miR-92b	-0,3	6,0E-01	9,9E-01
bta-miR-138	-1,0	5,9E-01	9,4E-01	bta-miR-183	-0,8	4,5E-01	8,3E-01	Novel:eca-miR-9040	1,0	6,0E-01	9,9E-01
Novel:X_45020	1,1	5,9E-01	9,4E-01	Novel:eca-miR-628a	1,5	4,5E-01	8,3E-01	Novel:6_39307	-0,4	6,1E-01	9,9E-01
Novel:eca-miR-138	-1,0	5,9E-01	9,4E-01	Novel:6_38564	0,8	4,5E-01	8,3E-01	Novel:20_20852	-0,5	6,1E-01	9,9E-01
bta-miR-128	0,3	6,0E-01	9,4E-01	bta-miR-181a	-0,8	4,5E-01	8,3E-01	bta-miR-126-5p	0,9	6,2E-01	9,9E-01

bta-miR-18a	0,8	6,0E-01	9,4E-01	bta-miR-371	-0,3	4,5E-01	8,3E-01	Novel:chi-miR-1307-5p	0,5	6,2E-01	9,9E-01
bta-miR-30e-5p	0,2	6,0E-01	9,4E-01	Novel:24_26327	1,8	4,5E-01	8,3E-01	bta-miR-502b	-0,4	6,2E-01	9,9E-01
Novel:29_29980	0,7	6,1E-01	9,4E-01	bta-miR-92b	-0,5	4,5E-01	8,3E-01	bta-miR-7858	1,0	6,2E-01	9,9E-01
Novel:28_29427	0,7	6,1E-01	9,4E-01	Novel:eca-miR-130b	-0,6	4,5E-01	8,3E-01	Novel:4_34592	-0,5	6,3E-01	9,9E-01
bta-miR-107	-0,3	6,1E-01	9,4E-01	Novel:eca-miR-101	-0,6	4,6E-01	8,3E-01	Novel:eca-miR-107b	-0,2	6,3E-01	9,9E-01
bta-miR-196a	0,4	6,2E-01	9,4E-01	bta-miR-150	1,5	4,7E-01	8,4E-01	bta-miR-30f	-0,8	6,3E-01	9,9E-01
bta-miR-431	-1,1	6,2E-01	9,4E-01	bta-miR-30f	-1,2	4,7E-01	8,4E-01	bta-miR-29a	-0,8	6,3E-01	9,9E-01
bta-miR-30a-5p	-0,2	6,3E-01	9,4E-01	bta-miR-27b	0,3	4,7E-01	8,4E-01	bta-miR-339b	1,1	6,4E-01	9,9E-01
bta-miR-2397-3p	-1,1	6,4E-01	9,4E-01	bta-miR-98	-1,2	4,7E-01	8,4E-01	bta-miR-2284x	0,1	6,4E-01	9,9E-01
Novel:eca-miR-130b	-0,4	6,5E-01	9,4E-01	bta-miR-2284ab	-0,5	4,7E-01	8,4E-01	Novel:eca-miR-26a	-0,3	6,5E-01	9,9E-01
bta-miR-331-3p	0,4	6,5E-01	9,4E-01	Novel:X_45020	1,1	4,8E-01	8,4E-01	Novel:16_11586	1,1	6,5E-01	9,9E-01
bta-miR-2285b	0,5	6,5E-01	9,4E-01	bta-miR-21-3p	0,9	4,8E-01	8,4E-01	Novel:eca-miR-1301	-0,9	6,5E-01	9,9E-01
bta-miR-146a	0,2	6,5E-01	9,4E-01	Novel:3_32033	1,2	4,8E-01	8,4E-01	bta-miR-197	0,8	6,5E-01	9,9E-01
Novel:18_16297	-0,6	6,5E-01	9,4E-01	bta-miR-2285g	0,7	4,9E-01	8,4E-01	bta-miR-6517	0,8	6,6E-01	9,9E-01
bta-miR-769	0,2	6,5E-01	9,4E-01	bta-miR-767	0,4	4,9E-01	8,4E-01	Novel:17_14163	-0,9	6,6E-01	9,9E-01
bta-miR-371	-0,2	6,5E-01	9,4E-01	bta-miR-345-3p	-1,2	4,9E-01	8,4E-01	bta-miR-146a	-0,2	6,6E-01	9,9E-01
bta-miR-455-5p	0,8	6,5E-01	9,4E-01	bta-miR-9-5p	0,8	4,9E-01	8,4E-01	bta-miR-6523a	0,4	6,6E-01	9,9E-01
Novel:8_41794	0,8	6,5E-01	9,4E-01	Novel:eca-miR-767-5p	0,4	4,9E-01	8,4E-01	bta-let-7f	-0,2	6,6E-01	9,9E-01
bta-miR-767	0,3	6,6E-01	9,4E-01	Novel:eca-miR-186	-0,6	5,0E-01	8,4E-01	Novel:13_7099	0,9	6,7E-01	9,9E-01
Novel:eca-miR-767-5p	0,3	6,6E-01	9,4E-01	bta-let-7c	1,2	5,0E-01	8,4E-01	bta-miR-431	0,9	6,7E-01	9,9E-01
Novel:eca-miR-194	0,4	6,6E-01	9,4E-01	bta-miR-186	-0,6	5,0E-01	8,4E-01	bta-miR-2484	0,7	6,7E-01	9,9E-01
bta-miR-191	-0,2	6,9E-01	9,5E-01	bta-miR-302b	-1,2	5,0E-01	8,4E-01	Novel:23_25542	-0,9	6,8E-01	9,9E-01
bta-miR-301a	-0,3	6,9E-01	9,5E-01	bta-miR-378c	-0,4	5,1E-01	8,4E-01	bta-miR-2446	0,9	6,8E-01	9,9E-01
bta-miR-1296	0,9	6,9E-01	9,5E-01	bta-miR-155	0,5	5,1E-01	8,4E-01	Novel:eca-miR-19a	0,6	6,8E-01	9,9E-01
Novel:23_24587	0,6	7,0E-01	9,5E-01	Novel:15_11311	-1,1	5,2E-01	8,5E-01	bta-miR-302c	-0,9	6,9E-01	9,9E-01
Novel:23_23280	0,6	7,0E-01	9,5E-01	bta-miR-6517	-1,2	5,2E-01	8,5E-01	bta-miR-132	0,8	6,9E-01	9,9E-01
bta-miR-130a	0,4	7,0E-01	9,5E-01	Novel:17_14702	-1,4	5,2E-01	8,5E-01	Novel:eca-miR-146b-5p	-0,6	6,9E-01	9,9E-01
Novel:15_10278	0,8	7,0E-01	9,5E-01	bta-miR-2435	1,1	5,3E-01	8,5E-01	Novel:eca-miR-199a-3p	-0,8	6,9E-01	9,9E-01

bta-miR-99b	-0,4	7,1E-01	9,5E-01	bta-miR-194	-0,4	5,3E-01	8,5E-01	Novel:15_10413	0,6	7,0E-01	9,9E-01
Novel:eca-miR-299	-0,8	7,1E-01	9,5E-01	bta-miR-411a	-0,8	5,3E-01	8,5E-01	Novel:28_29435	-0,3	7,0E-01	9,9E-01
bta-miR-1306	-0,8	7,1E-01	9,5E-01	bta-miR-210	0,6	5,3E-01	8,5E-01	bta-miR-130b	0,3	7,0E-01	9,9E-01
bta-miR-16a	0,4	7,1E-01	9,5E-01	bta-miR-2284aa	1,4	5,3E-01	8,5E-01	bta-miR-15b	0,4	7,0E-01	9,9E-01
Novel:eca-miR-15b	0,4	7,1E-01	9,5E-01	bta-miR-301a	-0,4	5,3E-01	8,5E-01	bta-miR-708	-0,8	7,0E-01	9,9E-01
bta-miR-101	0,2	7,2E-01	9,5E-01	bta-miR-769	-0,6	5,4E-01	8,5E-01	bta-let-7d	0,6	7,0E-01	9,9E-01
Novel:eca-miR-107b	-0,2	7,2E-01	9,5E-01	Novel:19_16745	0,8	5,4E-01	8,5E-01	bta-miR-2284p	0,8	7,1E-01	9,9E-01
Novel:6_36914	-0,6	7,3E-01	9,5E-01	bta-miR-152	1,3	5,4E-01	8,5E-01	Novel:19_17440	0,8	7,1E-01	9,9E-01
bta-miR-30d	0,1	7,4E-01	9,5E-01	Novel:6_37493	0,4	5,5E-01	8,5E-01	bta-miR-7	-0,8	7,1E-01	9,9E-01
bta-miR-1298	0,6	7,4E-01	9,5E-01	bta-miR-502b	0,7	5,5E-01	8,5E-01	Novel:27_28768	0,2	7,1E-01	9,9E-01
Novel:eca-miR-1298	0,6	7,4E-01	9,5E-01	Novel:4_34799	1,3	5,5E-01	8,5E-01	bta-miR-302b	-0,9	7,1E-01	9,9E-01
bta-miR-193b	0,7	7,5E-01	9,5E-01	bta-miR-130b	-0,4	5,5E-01	8,5E-01	bta-miR-2336	0,3	7,1E-01	9,9E-01
bta-miR-2346	-0,4	7,5E-01	9,5E-01	bta-miR-2346	-0,7	5,6E-01	8,6E-01	bta-miR-196a	0,3	7,2E-01	9,9E-01
bta-miR-34c	-0,2	7,5E-01	9,5E-01	bta-miR-191	-0,1	5,6E-01	8,6E-01	bta-miR-24-3p	-0,5	7,2E-01	9,9E-01
bta-miR-2318	0,5	7,5E-01	9,5E-01	bta-miR-222	0,4	5,6E-01	8,6E-01	bta-miR-381	-0,3	7,2E-01	9,9E-01
bta-miR-30c	0,1	7,5E-01	9,5E-01	bta-miR-1296	1,0	5,7E-01	8,6E-01	bta-miR-31	-0,4	7,2E-01	9,9E-01
bta-miR-105b	-0,7	7,5E-01	9,5E-01	bta-miR-451	-0,9	5,8E-01	8,7E-01	Novel:eca-miR-31	-0,4	7,2E-01	9,9E-01
Novel:7_39572	0,6	7,6E-01	9,5E-01	Novel:5_35327	1,0	5,8E-01	8,7E-01	bta-miR-92a	0,7	7,3E-01	9,9E-01
Novel:eca-miR-105	-0,7	7,6E-01	9,5E-01	bta-miR-497	-1,2	5,8E-01	8,7E-01	Novel:1_591	-0,8	7,3E-01	9,9E-01
bta-miR-2399-5p	-0,6	7,6E-01	9,5E-01	bta-miR-3432a	-0,4	5,8E-01	8,7E-01	bta-miR-2285c	0,6	7,3E-01	9,9E-01
bta-miR-200c	0,5	7,6E-01	9,5E-01	Novel:14_9885	0,9	5,8E-01	8,7E-01	Novel:chi-miR-2284d	-0,4	7,4E-01	9,9E-01
Novel:20_20831	-0,3	7,6E-01	9,5E-01	bta-miR-2411-3p	-1,1	5,9E-01	8,7E-01	bta-miR-2419-3p	0,7	7,4E-01	9,9E-01
bta-miR-215	-0,5	7,7E-01	9,5E-01	bta-miR-17-3p	0,9	5,9E-01	8,8E-01	bta-miR-19b	0,3	7,4E-01	9,9E-01
bta-miR-26b	-0,2	7,7E-01	9,5E-01	bta-miR-378	-0,5	6,0E-01	8,9E-01	Novel:15_11314	-0,8	7,4E-01	9,9E-01
bta-miR-2435	-0,6	7,7E-01	9,5E-01	bta-miR-99b	0,5	6,1E-01	9,0E-01	bta-miR-25	0,3	7,4E-01	9,9E-01
Novel:5_35327	-0,6	7,7E-01	9,5E-01	bta-miR-2284v	1,1	6,2E-01	9,0E-01	Novel:4_34799	-0,6	7,5E-01	9,9E-01
bta-miR-6120-3p	-0,4	7,8E-01	9,5E-01	bta-miR-6119-3p	0,8	6,3E-01	9,1E-01	bta-miR-1260b	0,5	7,5E-01	9,9E-01
bta-miR-22-3p	0,1	7,8E-01	9,5E-01	Novel:eca-miR-26a	-0,3	6,3E-01	9,1E-01	Novel:eca-miR-7	-0,7	7,5E-01	9,9E-01
bta-miR-126-3p	-0,6	7,8E-01	9,5E-01	bta-miR-302c	-1,1	6,4E-01	9,1E-01	bta-miR-150	-0,7	7,5E-01	9,9E-01
Novel:12_5433	-0,2	7,9E-01	9,5E-01	Novel:15_11027	-0,7	6,4E-01	9,1E-01	bta-miR-215	-0,6	7,6E-01	9,9E-01
bta-miR-411c-5p	0,5	7,9E-01	9,5E-01	bta-miR-532	-0,5	6,4E-01	9,2E-01	bta-miR-592	0,7	7,6E-01	9,9E-01
bta-miR-28	0,2	8,0E-01	9,5E-01	Novel:6_36914	0,7	6,4E-01	9,2E-01	bta-miR-504	-0,7	7,6E-01	9,9E-01

bta-miR-2285t	-0,6	8,0E-01	9,5E-01	bta-miR-652	-0,7	6,6E-01	9,2E-01	bta-miR-455-5p	0,6	7,6E-01	9,9E-01
bta-miR-221	0,5	8,0E-01	9,5E-01	bta-miR-30b-5p	-0,5	6,6E-01	9,2E-01	Novel:8_41794	0,6	7,6E-01	9,9E-01
bta-miR-130b	-0,2	8,0E-01	9,5E-01	bta-miR-146a	0,5	6,6E-01	9,2E-01	bta-miR-26a	-0,3	7,7E-01	9,9E-01
bta-miR-2284z	0,2	8,1E-01	9,5E-01	bta-miR-1248	0,8	6,6E-01	9,2E-01	bta-miR-378	0,6	7,7E-01	9,9E-01
bta-miR-6517	-0,4	8,1E-01	9,5E-01	Novel:eca-miR-30c	-0,5	6,6E-01	9,2E-01	Novel:eca-miR-8984	0,4	7,7E-01	9,9E-01
Novel:eca-miR-22	0,1	8,1E-01	9,5E-01	bta-miR-26b	-0,4	6,6E-01	9,2E-01	bta-miR-105a	-0,4	7,7E-01	9,9E-01
Novel:4_34799	0,5	8,1E-01	9,5E-01	Novel:15_11314	0,9	6,7E-01	9,3E-01	bta-miR-652	-0,5	7,7E-01	9,9E-01
Novel:27_28768	-0,2	8,1E-01	9,5E-01	bta-miR-27a-3p	-0,4	6,7E-01	9,3E-01	Novel:6_37493	0,3	7,8E-01	9,9E-01
bta-miR-374a	-0,5	8,2E-01	9,5E-01	bta-miR-411c-5p	-0,7	6,7E-01	9,3E-01	Novel:7_39799	-0,6	7,8E-01	9,9E-01
bta-miR-877	-0,3	8,2E-01	9,6E-01	Novel:13_7099	-0,8	6,7E-01	9,3E-01	Novel:21_21851	0,6	7,8E-01	9,9E-01
Novel:14_9885	-0,4	8,2E-01	9,6E-01	Novel:chi-miR-1307-5p	-0,4	6,8E-01	9,3E-01	Novel:7_39572	0,6	7,8E-01	9,9E-01
bta-miR-378	0,1	8,3E-01	9,6E-01	bta-miR-149-5p	-0,5	6,8E-01	9,3E-01	Novel:eca-miR-130b	0,2	7,9E-01	9,9E-01
Novel:eca-miR-340-5p	-0,2	8,3E-01	9,6E-01	bta-miR-93	-0,4	6,8E-01	9,3E-01	bta-miR-2387	0,7	7,9E-01	9,9E-01
bta-miR-2904	0,2	8,4E-01	9,6E-01	bta-miR-126-5p	-0,7	6,9E-01	9,3E-01	bta-miR-2285l	-0,6	8,0E-01	9,9E-01
bta-miR-2284y	0,2	8,4E-01	9,6E-01	bta-miR-484	-0,3	6,9E-01	9,3E-01	bta-miR-143	-0,2	8,0E-01	9,9E-01
bta-miR-532	-0,1	8,4E-01	9,6E-01	bta-miR-432	-0,8	6,9E-01	9,3E-01	bta-miR-107	-0,1	8,0E-01	9,9E-01
bta-miR-194	0,1	8,5E-01	9,6E-01	bta-miR-449c	-0,8	7,0E-01	9,3E-01	Novel:eca-miR-21	-0,2	8,0E-01	9,9E-01
bta-miR-339b	0,4	8,6E-01	9,6E-01	bta-miR-18a	-0,7	7,0E-01	9,3E-01	Novel:13_7321	-0,6	8,0E-01	9,9E-01
Novel:eca-miR-101	0,1	8,6E-01	9,6E-01	bta-miR-425-3p	0,8	7,0E-01	9,3E-01	bta-miR-21-5p	-0,2	8,0E-01	9,9E-01
bta-miR-184	-0,1	8,6E-01	9,6E-01	bta-miR-19b	0,3	7,0E-01	9,3E-01	bta-miR-380-3p	0,3	8,0E-01	9,9E-01
bta-miR-19a	0,2	8,6E-01	9,6E-01	bta-miR-339b	-0,7	7,0E-01	9,3E-01	Novel:eca-miR-380	0,3	8,0E-01	9,9E-01
bta-miR-6529a	0,1	8,6E-01	9,6E-01	bta-miR-2318	-0,6	7,1E-01	9,3E-01	bta-miR-129-5p	0,4	8,2E-01	9,9E-01
bta-miR-2419-5p	-0,1	8,7E-01	9,6E-01	bta-miR-106b	-0,5	7,1E-01	9,3E-01	bta-miR-129	0,4	8,2E-01	9,9E-01
bta-miR-378c	0,1	8,7E-01	9,6E-01	Novel:eca-miR-19a	-0,5	7,2E-01	9,3E-01	bta-miR-151-5p	-0,1	8,2E-01	9,9E-01
bta-miR-378b	0,1	8,7E-01	9,6E-01	bta-miR-2285k	0,3	7,2E-01	9,3E-01	bta-miR-16b	0,1	8,2E-01	9,9E-01
bta-miR-502b	0,2	8,9E-01	9,7E-01	bta-miR-30e-5p	0,1	7,3E-01	9,3E-01	bta-miR-425-5p	0,1	8,3E-01	9,9E-01
bta-miR-338	0,3	8,9E-01	9,7E-01	bta-miR-132	-0,7	7,3E-01	9,3E-01	Novel:eca-miR-143	0,2	8,3E-01	9,9E-01
Novel:28_29435	-0,1	8,9E-01	9,7E-01	bta-miR-2419-5p	-0,2	7,3E-01	9,3E-01	Novel:27_28600	-0,4	8,3E-01	9,9E-01
Novel:eca-miR-8984	0,2	8,9E-01	9,7E-01	Novel:eca-miR-323-3p	0,7	7,3E-01	9,3E-01	bta-miR-2284ac	-0,4	8,3E-01	9,9E-01
bta-miR-345-3p	-0,2	9,0E-01	9,7E-01	bta-miR-323	0,7	7,3E-01	9,3E-01	bta-miR-1842	-0,5	8,3E-01	9,9E-01

Novel:15_10413	0,2	9,0E-01	9,8E-01	bta-miR-1185	-0,7	7,3E-01	9,3E-01	bta-miR-133a	0,2	8,3E-01	9,9E-01
bta-miR-125b	-0,2	9,2E-01	9,8E-01	bta-miR-105b	0,6	7,4E-01	9,3E-01	Novel:19_16745	0,2	8,3E-01	9,9E-01
bta-miR-126-5p	0,1	9,2E-01	9,8E-01	Novel:eca-miR-105	0,6	7,4E-01	9,3E-01	bta-miR-1246	-0,1	8,3E-01	9,9E-01
bta-miR-425-5p	0,1	9,3E-01	9,8E-01	bta-miR-101	-0,2	7,5E-01	9,4E-01	Novel:X_44451	-0,4	8,3E-01	9,9E-01
bta-miR-103	0,0	9,3E-01	9,8E-01	Novel:27_28768	-0,4	7,5E-01	9,4E-01	bta-miR-449a	-0,2	8,4E-01	9,9E-01
bta-miR-874	-0,1	9,3E-01	9,8E-01	bta-miR-2483-5p	0,7	7,6E-01	9,4E-01	bta-miR-744	0,1	8,4E-01	9,9E-01
bta-miR-105a	-0,1	9,3E-01	9,8E-01	Novel:eca-miR-299	-0,6	7,6E-01	9,4E-01	Novel:eca-miR-184	-0,1	8,4E-01	9,9E-01
bta-miR-132	0,2	9,3E-01	9,8E-01	Novel:chi-miR-3432-5p	-0,3	7,6E-01	9,4E-01	bta-miR-423-5p	0,3	8,5E-01	9,9E-01
bta-miR-339a	-0,1	9,4E-01	9,8E-01	bta-miR-16b	0,3	7,6E-01	9,4E-01	bta-miR-210	0,1	8,5E-01	9,9E-01
bta-miR-6523a	0,1	9,4E-01	9,8E-01	Novel:20_20831	-0,3	7,6E-01	9,4E-01	bta-miR-2419-5p	0,2	8,6E-01	9,9E-01
bta-miR-34b	-0,1	9,5E-01	9,8E-01	bta-miR-2299-5p	0,6	7,7E-01	9,5E-01	bta-miR-375	0,2	8,6E-01	9,9E-01
Novel:27_28600	-0,1	9,6E-01	9,8E-01	bta-miR-34c	0,3	7,8E-01	9,5E-01	bta-miR-125b	-0,3	8,6E-01	9,9E-01
bta-miR-2284ac	-0,1	9,6E-01	9,8E-01	bta-miR-6523a	-0,3	7,8E-01	9,5E-01	bta-miR-30e-5p	0,2	8,6E-01	9,9E-01
Novel:eca-miR-19a	0,1	9,6E-01	9,8E-01	bta-miR-184	0,3	7,8E-01	9,5E-01	bta-miR-30d	0,2	8,6E-01	9,9E-01
Novel:13_7099	-0,1	9,6E-01	9,8E-01	bta-miR-302d	-0,4	7,9E-01	9,6E-01	bta-miR-2284v	0,3	8,7E-01	9,9E-01
bta-miR-1307	0,0	9,6E-01	9,8E-01	bta-miR-181d	-0,5	7,9E-01	9,6E-01	bta-miR-126-3p	0,4	8,7E-01	9,9E-01
bta-miR-30b-3p	-0,1	9,7E-01	9,8E-01	Novel:eca-miR-184	0,2	8,0E-01	9,6E-01	bta-miR-30b-5p	-0,2	8,7E-01	9,9E-01
bta-miR-21-3p	0,1	9,7E-01	9,8E-01	Novel:28_29435	0,3	8,0E-01	9,6E-01	Novel:eca-miR-30c	-0,2	8,7E-01	9,9E-01
Novel:eca-miR-184	0,0	9,7E-01	9,8E-01	Novel:15_10413	-0,4	8,0E-01	9,6E-01	bta-miR-301a	0,2	8,7E-01	9,9E-01
bta-miR-2284x	0,0	9,7E-01	9,8E-01	bta-miR-15b	0,2	8,0E-01	9,6E-01	bta-miR-339a	-0,1	8,7E-01	9,9E-01
bta-miR-380-3p	0,0	9,8E-01	9,8E-01	bta-miR-105a	0,3	8,2E-01	9,7E-01	bta-miR-30a-5p	-0,1	8,7E-01	9,9E-01
Novel:eca-miR-380	0,0	9,8E-01	9,8E-01	Novel:21_21202	0,4	8,2E-01	9,7E-01	bta-miR-371	0,1	8,7E-01	9,9E-01
bta-miR-140	0,0	9,9E-01	9,9E-01	bta-miR-1298	0,4	8,2E-01	9,7E-01	bta-miR-3660	-0,3	8,8E-01	9,9E-01
Novel:chi-miR-1307-5p	0,0	9,9E-01	9,9E-01	Novel:eca-miR-1298	0,4	8,2E-01	9,7E-01	Novel:3_32033	0,2	8,8E-01	9,9E-01
				bta-miR-2446	0,5	8,3E-01	9,7E-01	Novel:eca-miR-148a	0,1	8,9E-01	9,9E-01
				Novel:eca-miR-9040	0,4	8,3E-01	9,7E-01	Novel:7_39472	0,3	9,0E-01	9,9E-01
				bta-miR-877	-0,3	8,3E-01	9,7E-01	Novel:6_38564	-0,1	9,0E-01	9,9E-01
				bta-miR-151-5p	-0,2	8,3E-01	9,7E-01	Novel:eca-miR-100	-0,3	9,0E-01	9,9E-01
				Novel:16_12359	-0,4	8,4E-01	9,7E-01	bta-miR-100	-0,3	9,0E-01	9,9E-01
				Novel:27_28600	0,3	8,4E-01	9,7E-01	bta-miR-34b	0,2	9,0E-01	9,9E-01

bta-miR-2284ac	0,3	8,4E-01	9,7E-01	Novel:9_43930	0,3	9,0E-01	9,9E-01
bta-miR-455-5p	0,3	8,4E-01	9,7E-01	Novel:6_36682	-0,1	9,0E-01	9,9E-01
Novel:8_41794	0,3	8,4E-01	9,7E-01	bta-miR-2299-5p	0,3	9,0E-01	9,9E-01
Novel:eca-miR-221	0,2	8,5E-01	9,7E-01	bta-miR-148b	0,1	9,1E-01	9,9E-01
bta-miR-27a-5p	-0,4	8,6E-01	9,7E-01	bta-miR-6518	0,2	9,1E-01	9,9E-01
bta-miR-23b-3p	-0,2	8,6E-01	9,7E-01	Novel:15_10465	0,2	9,1E-01	9,9E-01
bta-miR-2285b	-0,2	8,6E-01	9,7E-01	bta-miR-2284aa	-0,2	9,2E-01	9,9E-01
bta-miR-196a	0,1	8,6E-01	9,7E-01	Novel:eca-miR-299	-0,2	9,2E-01	9,9E-01
Novel:23_24587	-0,3	8,7E-01	9,7E-01	bta-miR-1298	0,2	9,2E-01	9,9E-01
Novel:23_23280	-0,3	8,7E-01	9,7E-01	Novel:eca-miR-1298	0,2	9,2E-01	9,9E-01
bta-miR-339a	0,2	8,7E-01	9,7E-01	Novel:X_44463	0,1	9,2E-01	9,9E-01
bta-miR-34b	-0,2	8,7E-01	9,7E-01	bta-miR-15a	-0,1	9,2E-01	9,9E-01
bta-miR-30d	0,0	8,9E-01	9,8E-01	bta-miR-103	0,0	9,2E-01	9,9E-01
Novel:1_591	-0,3	8,9E-01	9,8E-01	bta-miR-2483-5p	-0,2	9,3E-01	9,9E-01
bta-miR-410	-0,2	8,9E-01	9,8E-01	bta-miR-1343-3p	0,1	9,3E-01	9,9E-01
bta-miR-125b	0,2	8,9E-01	9,8E-01	bta-miR-6119-5p	0,0	9,3E-01	9,9E-01
bta-miR-381	-0,1	8,9E-01	9,8E-01	Novel:X_44441	-0,1	9,3E-01	9,9E-01
bta-miR-181b	-0,2	9,0E-01	9,8E-01	bta-miR-874	-0,1	9,3E-01	9,9E-01
bta-miR-30a-5p	-0,1	9,0E-01	9,8E-01	Novel:X_45065	0,0	9,3E-01	9,9E-01
bta-miR-199a-5p	0,2	9,1E-01	9,8E-01	bta-miR-424-3p	-0,1	9,4E-01	9,9E-01
bta-miR-3660	0,2	9,1E-01	9,8E-01	bta-miR-660	-0,1	9,4E-01	9,9E-01
bta-miR-107	-0,1	9,1E-01	9,8E-01	Novel:2_19965	0,1	9,4E-01	9,9E-01
bta-miR-421	0,1	9,2E-01	9,8E-01	Novel:eca-miR-9087	0,2	9,4E-01	9,9E-01
bta-let-7i	0,1	9,2E-01	9,8E-01	Novel:eca-miR-340-5p	-0,1	9,4E-01	9,9E-01
bta-miR-378d	0,2	9,2E-01	9,8E-01	Novel:eca-miR-129a-5p	-0,1	9,4E-01	9,9E-01
bta-miR-302a	-0,2	9,2E-01	9,8E-01	Novel:oar-miR-323c	-0,1	9,4E-01	9,9E-01
bta-miR-128	-0,1	9,3E-01	9,8E-01	bta-miR-199a-5p	-0,1	9,5E-01	9,9E-01
bta-miR-380-3p	-0,1	9,3E-01	9,8E-01	bta-miR-2887	0,1	9,6E-01	9,9E-01
Novel:eca-miR-380	-0,1	9,3E-01	9,8E-01	Novel:eca-miR-450a	0,1	9,6E-01	9,9E-01
bta-miR-17-5p	-0,1	9,3E-01	9,8E-01	bta-miR-450a	0,1	9,6E-01	9,9E-01

bta-miR-6529a	-0,1	9,4E-01	9,8E-01	bta-miR-1296	0,1	9,6E-01	9,9E-01
Novel:eca-miR-340-5p	-0,1	9,4E-01	9,8E-01	Novel:20_20831	0,0	9,6E-01	9,9E-01
Novel:15_10278	-0,2	9,4E-01	9,8E-01	bta-miR-767	0,0	9,6E-01	9,9E-01
Novel:eca-miR-194	-0,1	9,4E-01	9,8E-01	Novel:eca-miR-767-5p	0,0	9,6E-01	9,9E-01
bta-miR-103	0,1	9,4E-01	9,8E-01	bta-miR-192	0,1	9,6E-01	9,9E-01
Novel:eca-miR-107b	0,1	9,5E-01	9,9E-01	Novel:eca-miR-192	0,1	9,7E-01	9,9E-01
bta-miR-30c	-0,1	9,6E-01	9,9E-01	bta-miR-149-5p	0,0	9,7E-01	9,9E-01
bta-miR-2344	0,1	9,6E-01	9,9E-01	bta-miR-9-5p	0,0	9,7E-01	9,9E-01
bta-miR-425-5p	0,0	9,6E-01	9,9E-01	bta-miR-877	0,0	9,8E-01	9,9E-01
bta-miR-215	-0,1	9,6E-01	9,9E-01	Novel:eca-miR-22	0,0	9,8E-01	9,9E-01
bta-miR-874	0,1	9,7E-01	9,9E-01	bta-miR-10b	-0,1	9,8E-01	9,9E-01
bta-miR-125a	0,0	9,7E-01	9,9E-01	bta-miR-6123	0,0	9,8E-01	9,9E-01
bta-miR-30b-3p	0,1	9,7E-01	9,9E-01	bta-miR-2885	0,0	9,8E-01	9,9E-01
bta-miR-6524	-0,1	9,8E-01	9,9E-01	Novel:X_45020	0,0	9,8E-01	9,9E-01
bta-miR-340	0,0	9,8E-01	9,9E-01	bta-miR-22-3p	0,0	9,9E-01	9,9E-01
Novel:7_39572	0,0	9,8E-01	9,9E-01	bta-miR-2285q	0,0	9,9E-01	9,9E-01
Novel:eca-miR-8984	0,0	9,9E-01	1,0E+00	Novel:chi-miR-374a-3p	0,0	9,9E-01	9,9E-01
bta-miR-20a	0,0	9,9E-01	1,0E+00	bta-miR-30b-3p	0,0	9,9E-01	9,9E-01
bta-miR-454	0,0	9,9E-01	1,0E+00	bta-miR-191	0,0	9,9E-01	9,9E-01
bta-miR-2284x	0,0	1,0E+00	1,0E+00	bta-miR-148a	0,0	1,0E+00	1,0E+00

Supplementary File S3. Comparison of sequencing data and quantitative Real Time PCR (qPCR) using two reference smallRNA (SNORD95 and RNU6) for five differentially expressed DE-miRNAs (let-7a-5p, miR-130a, miR-34a, miR-423-5p and miR-486) in three groups (*in vivo*, *in vitro*-CTR and *in vitro*+MVs). For miRNA sequencing data the fold change FC was calculated using the ratio between the average number of reads of triplicates within the groups and the average number of reads of *in vivo* group triplicates. For qPCR results, FC was calculated using 2^-ΔΔCt method.

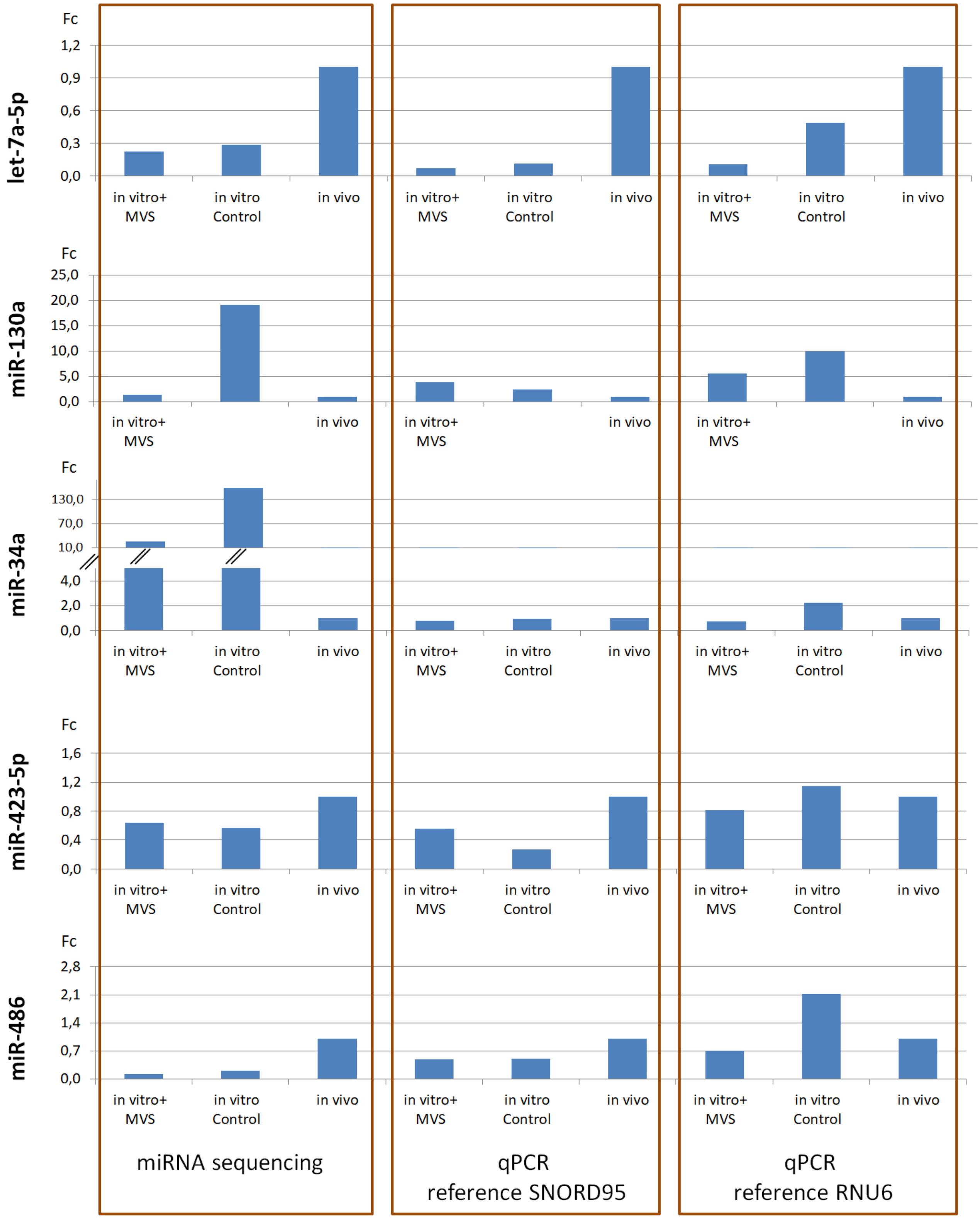

Supplement: Supplementary file 1 — Supplementary information. [file 41598_2019_57060_MOESM1_ESM.pdf]
